# Antibacterial and Antifungal Potential of *Helichrysum italicum* (Roth) G. Don Essential Oil

**DOI:** 10.3390/antibiotics13080722

**Published:** 2024-08-01

**Authors:** Olja Šovljanski, Milica Aćimović, Ana Tomić, Biljana Lončar, Ana Miljković, Ivana Čabarkapa, Lato Pezo

**Affiliations:** 1 Faculty of Technology Novi Sad, University of Novi Sad, Bulevar Cara Lazara 1, 21000 Novi Sad, Serbia; oljasovljanski@uns.ac.rs (O.Š.); anav@uns.ac.rs (A.T.); cbiljana@uns.ac.rs (B.L.); 2 Institute of Field and Vegetable Crops Novi Sad, Maksima Gorkog 30, 21000 Novi Sad, Serbia; milica.acimovic@ifvcns.ns.ac.rs; 3 Faculty of Medicine, University of Novi Sad, Hajduk Veljkova 3, 21000 Novi Sad, Serbia; ana.miljkovic@mf.uns.ac.rs; 4 Institute of Food Technology, University of Novi Sad, Bulevar Cara Lazara 1, 21000 Novi Sad, Serbia; ivana.cabarkapa@fins.uns.ac.rs; 5 Institute of General and Physical Chemistry, University of Belgrade, Studentski trg 12, 11000 Belgrade, Serbia

**Keywords:** immortelle, curry plant, biological activity

## Abstract

*Helichrysum italicum* (Roth) G. Don is a typical Mediterranean plant, with limited distribution on the islands of Sardinia, Corsica, and the Iberian Peninsula, as well as the islands of the Adriatic Sea and the Balkan Peninsula. In these regions, *H. italicum* is mainly collected from spontaneous nature, while in recent years, there has been a pronounced cultivation trend due to increased demand and market requirements for constant quality of raw materials. Bearing in mind that biological activity is linked with chemical composition, this review aimed to collect data from different scientific databases (Scopus, PubMed, Web of Science, and Google Scholar) on the antimicrobial activity of essential oil and its chemical composition. A total of 20 papers investigating the antibacterial, antibiofilm, and antifungal activities of *H. italicum* essential oil were found. Furthermore, in these samples, several compounds occurred as dominant: neryl acetate, α-pinene, and γ-curcumene. These compounds are known for their antimicrobial properties, which likely contribute to the essential oil’s efficacy against various microbial strains.

## 1. Introduction

The large genus *Helichrysum* includes over 500 species distributed worldwide [1]. However, as a complex species, *Helichrysum italicum* (Roth) G. Don (1830) is the best known and most widespread, but it expresses a high level of polymorphism in its morphological traits and chemical characteristics. Several subspecies of *H. italicum*, including *H. italicum* subsp. *italicum*, *H. italicum* subsp. *microphyllum*, and *H. italicum* subsp. *serotinum*, are well-known, each with distinct essential-oil compositions, contributing to variations in antimicrobial efficacy observed across different studies [2]. Additionally, the distribution of these subspecies often overlaps, which can further influence the chemical profiles and biological activities of the essential oils. Worldwide, *H. italicum* is a favorite ornamental plant, cut flower, traditional remedy, spice, cosmetic, and perfume ingredient [2,3]. It has been used since ancient times, among Romans and Greeks, as a decoction made from its flowering tops for topical application due to its exceptional wound-healing properties. It is especially effective for treating hematoma, scars and cuts, and chronic inflammatory skin diseases such as eczema and psoriasis. Additionally, it is used for treating allergy-related ailments such as conjunctivitis and hay fever [4]. In the contemporary cosmetic era, *H. italicum* serves as an active and fragrance compound in phyto-derived formulations for treating and protecting delicate and irritated skin after sunburn and erythema [5]. Furthermore, recent trend research suggests that the internal application of *H. italicum* also has scientific validation [6]. In addition, the researchers’ focus is also on the antimicrobial potential of this plant, which is indicated by a large number of scientific articles.

On the other hand, antimicrobial resistance (AMR) is a significant global health concern, undermining the effectiveness of antibiotics and complicating the treatment of infections. The overuse and misuse of antibiotics in healthcare, agriculture, and animal husbandry have accelerated the development and spread of resistant microorganisms. This growing resistance challenges the management of infectious diseases and stresses healthcare systems worldwide. The search for new antimicrobial agents has led researchers to explore natural products, particularly essential oils from medicinal plants, due to their diverse bioactive compounds. *H. italicum* essential oil has shown significant antimicrobial activity against foodborne pathogens such as *Escherichia coli*, *Salmonella* Typhimurium, and *Listeria monocytogenes*. These pathogens are known for causing severe gastrointestinal illnesses through contaminated food. The essential oil’s mechanism of action includes disrupting bacterial cell membranes and interfering with metabolic processes, making it a promising candidate for enhancing food safety and preservation [7]. The targeted essential oil also exhibits antifungal properties, effective against plant pathogens like *Botrytis cinerea* and *Fusarium oxysporum*, which are responsible for considerable agricultural losses. The antifungal action is primarily due to the disruption of fungal cell walls and membranes, leading to cell death [8]. Beyond food and agriculture, *H. italicum* essential oil holds potential for clinical applications in addressing antibiotic-resistant infections. Its broad-spectrum antimicrobial activity positions it as a potential therapeutic agent against AMR. Additionally, its environmental friendliness and biodegradability make it an attractive alternative to synthetic antimicrobial agents in various applications.

This review explores the antimicrobial potential of *H. italicum* essential oil, evaluating its effectiveness against various pathogens, including those responsible for foodborne illnesses and plant diseases.

## 2. Review Methodology

This systematic review and a comprehensive literature search were conducted using the following databases: Scopus, PubMed, Web of Science, and Google Scholar. The search terms primarily included “*Helichrysum italicum*”, “essential oil”, “antimicrobial activity”, and “chemical composition”. Articles published up to July 2024 were considered. After the initial search, the additional search items included terms that were specific to the database, such as the type of sensitive microorganism, certain chemical compounds, etc. Studies were included if they met the following criteria: investigated the antimicrobial activity of *H. italicum* essential oil, reported on the chemical composition of the targeted essential oil, and published in peer-reviewed journals. Studies were excluded if they were not in English, did not provide sufficient data, or were reviews or meta-analyses themselves. Selection of data was performed independently by authors, using a standardized form. Extracted data included study design, antimicrobial testing methods, chemical composition of the essential oil, and outcomes.

## 3. Results

The current discussion on the antimicrobial activity of *H. italicum* essential oil is very relevant. By reviewing different scientific bases (Scopus, PubMed, Web of Science, and Google Scholar), 20 papers were found to investigate the antibacterial, antibiofilm, and antifungal activities of *H. italicum* essential oil (Table 1). Comparing the antimicrobial activity of the samples from this study to the findings from scientific bases on *H. italicum* essential oil, we observed several insightful parallels and contrasts. Table 1 summarizes various studies on the antimicrobial effects of *H. italicum* essential oil, highlighting its chemical composition and efficacy against various microbial strains using different methodologies. Namely, the studies reviewed consistently highlight neryl acetate, α-pinene, and γ-curcumene as key components of *H. italicum* essential oil. These compounds are known for their antimicrobial properties, which likely contribute to the essential oil’s efficacy against various microbial strains.

Table 1 highlights various antimicrobial tests used to assess the antimicrobial potential of the targeted essential oil. Each study employed specific antimicrobial testing methodologies, such as agar well diffusion, broth microdilution, and vapor phase assays, to assess the oil’s efficacy. The broth microdilution test is the most used antimicrobial test to analyze the antimicrobial potential of *H. italicum* oil. This test is a quantitative technique used to determine the Minimal Inhibitory Concentration (MIC) of antimicrobial agents. The test substance is serially diluted in a liquid growth medium and inoculated with the test microorganism. After incubation, the MIC is defined as the lowest concentration of the substance that inhibits visible microbial growth. Providing quantitative data, this test allows for the precise determination of MIC values, but it is also suitable for testing a wide range of microorganisms. At the same time, this procedure can be labor-intensive and time-consuming, and it requires careful control of experimental conditions to ensure reproducibility.

The lack of consistency in antimicrobial-assessment in vitro methods is noteworthy. The agar well-diffusion method is a simple and cost-effective qualitative technique for evaluating antimicrobial activity. Wells are created in an agar plate inoculated with the test microorganism, and the essential oil is introduced into the wells. After incubation, antimicrobial activity is determined by measuring the zone of inhibition around the wells. This method allows for a visual comparison of antimicrobial activity against multiple microorganisms but can be influenced by factors like the diffusion rate and solubility of the oil. Studies by Zeremski et al. [9], Servi and Servi [10], and Mollova et al. [15] used this method, reporting significant inhibition zones for various microorganisms. The vapor-phase assay evaluates the antimicrobial activity of volatile compounds by placing the test substance in a sealed environment with the microorganism. This method is suitable for essential oils used as fumigants or air disinfectants and minimizes direct contact. However, it has limitations, such as qualitative data and complex setups. Stupar et al. [25] used this assay to test *H. italicum* essential oil against fungi, highlighting its potential for air disinfection and preservation. Choosing the right methodology is crucial. Agar well diffusion shows clear inhibition zones but may not reflect MIC accurately, while broth microdilution provides precise MIC measurements. Combining multiple tests provides a robust understanding and a good base for in vivo studies [29].

Table 1 also shows that a total of 59 microorganisms were used in 20 papers dealing with the antimicrobial potential of *H. italicum* essential oil, of which the most represented was *S. aureus* (16 papers), followed by *E. coli* (15 papers) and *P. aeruginosa* (14 papers). The antimicrobial efficacy of *H. italicum* essential oil has been demonstrated in various studies employing different testing methodologies. Zeremski et al. [9] utilized agar well-diffusion and broth-microdilution methods to assess the oil’s activity, identifying *γ*-curcumene (13.11–19.98%), *α*-pinene (9.75–14.45%), and *ar*-curcumene (5.8–14.0%) as major components. Their tests showed significant antimicrobial activity against a range of bacteria, yeast, and fungi. Servi and Servi [10] also employed agar well-diffusion and broth-microdilution techniques, focusing on the contribution of the main oil’s constituents (neryl acetate (15.6%), *α*-pinene (11.5%), and *γ*-curcumene (11.3%)). Their study demonstrated the essential oil’s antimicrobial potential against *S. aureus*, *Bacillus subtilis*, *B. cereus*, *P. aeruginosa*, and *E. coli*. Similarly, Balazs et al. [11] conducted broth microdilution assays, identifying neryl acetate (21.2%) and *ar*-curcumene (15.9%) as key components, with antimicrobial effects against *P. aeruginosa*, *Streptococcus pneumoniae*, *Haemophilus influenzae*, and *Haemophilus parainfluenzae* [30]. Further studies, such as those by Weglarz et al. [12], emphasized the broad antimicrobial spectrum of *H. italicum* essential oil. Using broth microdilution, they highlighted the effectiveness of oil rich in neryl acetate (16.4–20.3%), nerol (4.5–15.7%), and *α*-pinene (4.1–10.4%) against *E. coli*, *P. aeruginosa*, and *S. aureus*. Aćimović et al. [13] also reported similar findings, noting *γ*-curcumene (13.6%), *β*-selinene (12.2%), and *α*-pinene (11.8%) as major constituents effective against a broad range of bacteria, including *E. coli*, *P. aeruginosa*, *Salmonella* Enteritidis, and *S*. Typhimurium.

Genčić et al. [14] investigated four different commercial samples of *H. italicum* essential oil from Bosnia and Herzegovina (21.3% of neryl acetate and 16.8% of *α*-pinene and *γ*-curcumene), Corsica (rich in neryl acetate, 35.0%; *β*-diketones, 11.9%; and *γ*-curcumene, 10.2%), and two samples from Serbia (4.9–19.9% *α*-pinene, 9.2–15.5% neryl acetate, and 10–12% *γ*-curcumene). The results of microdilution assay showed that antimicrobial efficiency against *Bacillus cereus*, *Enterococcus faecalis*, *Sarcina lutea*, *Staphylococcus aureus*, *S. epidermidis*, *Acinetobacter baumannii*, *Enterobacter aerogenes*, *E. coli*, S. enterica, and *C. albicans* is a result of synergistic activity between essential oil compounds (neryl acetate, *α*-pinene, *γ*- and *ar*-curcumenes, and *β*-diketones), and probably some minor constituents. Generally, the activity of *H. italicum* essential oil was better against Gram-positive bacteria in comparison to Gram-negative strains, while poor antifungal activity against *C. albicans* was noted.

Similarly, Mollova et al. [15] investigated the antimicrobial potential of two subspecies of *H. italicum* grown in Bulgaria (originating in Bosnia and France). The essential oil of *H. italicum* from France, rich in neryl acetate (33.9%) and *γ*-curcumen (8.8%) showed low antimicrobial activity against Gram-positive bacteria (*S. aureus* and *B. subtilis*), while all other microorganisms showed resistance to this essential oil. On the other hand, the sample from Bosnia rich in *α*-pinene (20.8%) and *γ*-curcumen (16.5%) did not show antimicrobial activity, except in the case of *A. brasiliensis*. Gram-negative bacteria used in this research were *E. coli*, *P. aeruginosa*, yeasts *S. cervisae* and *C. albicans*, and fungi *Aspergillus brasiliensis* and *Fusarium moniliforme*.

*H. italicum* essential oil from Montenegro, rich in *β*-eudesmene (21.7%) and *β*-bisabolene (19.9%), was tested against different strains of *S. aureus*, *E. coli*, *C. albicans*, *K. pneumoniae*, *A. baumanni*, and *P. aeruginosa* [16]. The results showed fungicidal activity against *C. albicans* and bactericidal potency against carbapenem-resistant *A. baumannii* at a concentration of 5%. In another study, conducted by Šćepanović et al. [18], Montenegrin *H. italicum* essential oil rich in *γ*-curcumene (14.1%), *β*-selinene (11.3%), and *ar*-curcumene (10.4%) was tested against nine bacteria and fungi (*E. coli*, *S. aureus*, *B. subtilis*, *S.* Enteritidis, *L. monocytogenes*, *P. mirabilis*, *P. aeruginosa*, *E. faecalis*, and *C. albicans*). The results showed that *S. aureus*, *L. monocytogenes*, and *B. subtilis* are very sensitive.

Dzamic et al. [17] investigated the antimicrobial activity of *H. italicum* essential oil of Croatian origin, rich in neryl acetate (20.4%) and *γ*-curcumene (14.1%), against 14 strains (*B. cereus*, *E. coli*, *L. monocytogenes*, *P. aeruginosa*, *S.* Typhimurium, *S. aureus*, *C. albicans*, *A. fumigatus*, *A. versicolor*, *A. ochraceus*, *A. niger*, *T. viride*, *P. funiculosum*, and *P. ochrochloron*). Using the microdilution method, MIC/MBC and MFC were concluded to have better antibacterial than antifungal activity. *H. italicum* essential oil of Croatian origin with neryl acetate (11.5%) and *α*-pinene (10.2%) was evaluated for antibacterial (*S. aureus*, *E. coli*, and *P. aeruginosa*) and antifungal (*C. albicans*) activities [26]. The results were more pronounced against *S. aureus* and *C. albicans*. Additionally, Malenica Staver et al. [19] stated that *H. italicum* essential oil rich in α-pinene and γ-curcumene (both present with 21.6%) expressed weak-to-moderate antimicrobial potential against tested bacteria and fungi, according to agar-well diffusion and microdilution assays. Conversely, the same essential oil sample is effective against *Mycobacterium avium* and *M. intracellulare* [20].

Algerian *H. italicum* essential oil with dominant *α*-cedrene (13.6%), *ar*-curcumene (11.4%), and geranyl acetate (10.1%) was tested for antimicrobial activity against 18 bacteria, yeasts, and fungi by agar diffusion method [21]. The results showed inhibition growth in all tested bacteria except *E. coli*, *K. pneumonia*, and *L. monocitogenes*, while fungi were most resistant to the oil. Moreover, endemic *H. italicum* var. *numidicum* from Algeria with isopropyl tetradecanoate (12.1%) and α-pinene (12.0%) as main compounds in essential oil was tested against six microorganisms [22]. The sample showed a modest effect against all tested bacteria (*Shigella* sp., *E. coli*, *K. pneumoniae*, *S. aureus*, and *Pseudomonas syringae*), but it had no effect on *C. albicans*.

*H. italicum* essential oil from France with neryl acetate (32.7–33.8%) and *γ*-curcumene (11.6–14.2%) successfully reduced the formation of *S. aureus* biofilms on different food-contact surfaces, and it inhibited the growth rate of surviving *E. coli* and *S. aureus* on vegetables [23,24].

The bacteriostatic activity of Greek *H. italicum* essential oil with dominant oxygenated monoterpenes geraniol (35.6%) and geranyl acetate (14.7%) was evaluated against *S. aureus*, *S. epidermidis*, *P. aeruginosa*, *K. pneumoniae*, *E. cloaceae*, and *E. coli* [28]. The results showed strong activity on *S. aureus* and *S. epidermidis*, weaker against *E. cloaceae*, *P. aeruginosa*, and *K. pneumoniae*, while the most resistant was *E. coli*.

The effectiveness of the *H. italicum* essential oil on 11 plant fungi was evaluated via a paper disk test in Petri dishes [27]. The fungi were *B. cinerea*, *C. beticola*, *F. oxysporum lycopersici*, *F. graminearum*, *H. oryzae*, *P. ultimum*, *P. oryzae*, *R. solani*, *S. rolfsii*, *P. capsici*, and *S. tritici.* According to the obtained results, *H. italicum* essential oil showed good antifungal activity against *P. ultimum and S. rolfsii* and moderate activity against *P. capsici* and *S. tritici*, indicating the possibility for employment as a biopesticide. Additionally, the antifungal activity of *H. italicum* essential oil was evaluated by micro-atmosphere methods in Petri plates against six fungi isolated from cultural heritage objects: *A. niger*, *A. ochraceus*, *B. spicifera*, *E. nigrum*, *Penicillium* sp., and *T. viride* [25]. The results showed that the most susceptible fungi to oil treatment were *E. nigrum* and *Penicillium* sp., as well as noted significant demelanizing activity against *A. niger*.

### 3.1. Gram-Positive Bacteria

#### 3.1.1. *Staphylococcus aureus*

*Staphylococcus aureus* is a Gram-positive bacterium that is a common cause of both hospital- and community-acquired infections. This pathogen is known for its ability to cause a range of illnesses, from minor skin infections to life-threatening diseases such as pneumonia, endocarditis, and sepsis. One of the major challenges in treating *S. aureus* infections is the bacterium’s propensity to develop resistance to multiple antibiotics, including methicillin, which has led to the emergence of methicillin-resistant *Staphylococcus aureus* (MRSA). MRSA infections are particularly difficult to treat and are associated with higher morbidity and mortality rates. Exploring alternative antimicrobial agents has become critical for this bacterium in this context. One such alternative is essential oils, which are known for their broad-spectrum antimicrobial properties such as *H. italicum* oil. Studies investigating the antimicrobial activity of *H. italicum* essential oil have shown promising results, particularly against *S. aureus*. The oil exhibits a significant inhibitory effect on the growth of this bacterium, which is attributed to the synergistic action of its various bioactive constituents. These compounds disrupt the bacterial cell membrane, leading to increased permeability and eventual cell death. Additionally, the oil’s components can interfere with essential bacterial enzymes and metabolic pathways, further enhancing its antimicrobial efficacy. On the other hand, antimicrobial resistance mechanisms in *S. aureus* are complex and multifaceted, involving the alteration of target sites and the production of antibiotic-degrading enzymes and efflux pumps that expel antibiotics from the bacterial cell. The use of *H. italicum* essential oil presents a novel approach to overcoming these resistance mechanisms. Unlike traditional antibiotics, essential oils comprise multiple active compounds that act through various mechanisms, making it more difficult for bacteria to develop resistance. Furthermore, the potential for synergy between different components of the oil may enhance its overall antimicrobial activity, reducing the likelihood of resistance development. The potential applications of *H. italicum* essential oil extend beyond its use as an unrelated antimicrobial agent. It could be integrated into topical formulations for the treatment of skin infections, incorporated into wound dressings to prevent infection, or used as a natural preservative in food and cosmetic products.

*H. italicum* essential oil, as an antimicrobial source against *S. aureus*, could be integrated into topical formulations for the treatment of skin infections, incorporated into wound dressings to prevent infection, or used as a natural preservative in food and cosmetic products. Additionally, its use in combination with conventional antibiotics could help restore the efficacy of these drugs against resistant strains of *S. aureus*, offering a complementary approach to existing antimicrobial therapies. In total, 16 papers dealing with the antimicrobial activity of *H. italicum* essential oil against *S. aureus* (Table 2). *H. italicum* essential oil has shown varying inhibition-zone diameters against *S. aureus* strains, reflecting its antimicrobial potency. Zeremski et al. [9] reported inhibition zones ranging from 10.00 to 19.67 mm for ATCC 25923, indicating moderate-to-strong activity. However, without specifying the concentration of the essential oil used, these measurements cannot be accurately interpreted in terms of antimicrobial activity. Only essential oils with activity concentrations below 100 µg/mL can be considered to have strong antimicrobial activity. Servi and Servi [10] observed a 10 mm inhibition zone for the same strain, while Mollova et al. [15] reported zones between 8 and 11.3 mm for ATCC 6538. Notably, Djihane et al. [21] found a significantly larger zone of 27 mm for ATCC 6538, highlighting exceptional antimicrobial activity.

Bouchaala et al. [22] and Malenica Staver et al. [19] observed inhibition zones of 10 mm and 8.5 mm for ATCC 25923, and 7.5 mm for MRSA, respectively. The MIC values for *H. italicum* essential oil demonstrate significant variability depending on the strain and oil composition. Servi and Servi [10] reported a MIC of 5 µg/mL for ATCC 25923, indicating potent inhibition. Weglarz et al. [12] found MIC values of 1–4 mg/mL for ATCC 25923 and 4–8 mg/mL for MRSA, suggesting that higher concentrations are needed for resistant strains. Aćimović et al. [13] reported a MIC > 454.5 µg/mL for ATCC 25923, indicating lower efficacy. This cut-off value is extremely low and maybe does not provide a precise measurement of the MIC. The actual MIC could be much higher, which could impact the interpretation of the antimicrobial efficacy of the essential oil. Genčić et al. [14] observed a MIC range of 0.30–10.00 µg/mL for ATCC 6538, while Oliva et al. [16] reported a MIC > 5% for ATCC 29213. Dzamic et al. [17] and Šćepanović et al. [18] reported MIC values of 15 mg/mL for ATCC 6538 and <1.4 µg/mL for ATCC 25923, respectively. Malenica Staver et al. [19] found MICs of 1.6 mg/mL for ATCC 25923 and 6.4 mg/mL for MRSA, while Cui et al. [23] and Cui et al. [24] reported MIC values of 0.5 mg/mL and 0.05% for ATCC 25923, respectively. Chinou et al. [28] found a MIC of 3.25 mg/mL for ATCC 25923. On the other hand, the MBC values provide insight into the concentration required to kill *S. aureus* cells. Weglarz et al. [12] reported MBCs of 16 mg/mL for ATCC 25923 and 32 mg/mL for MRSA, indicating the oil’s bactericidal properties. Aćimović et al. [13] found an MBC > 454.5 µg/mL for ATCC 25923, suggesting lower bactericidal activity. Dzamic et al. [17] observed an MBC > 15 mg/mL for ATCC 6538, while Šćepanović et al. [18] reported an MBC of 1.4 µg/mL for ATCC 25923, highlighting strong bactericidal activity. Malenica Staver et al. [19] found MBCs of 3.2 mg/mL for ATCC 25923 and 12.8 mg/mL for MRSA. Oliva et al. [16] reported an MBC > 5% for ATCC 29213, and Cui et al. [23] found an MBC of 1.00 mg/mL for ATCC 25923. Cui et al. [24] reported an MBC of 0.05% for the same strain.

These variations in antimicrobial activity of *H. italicum* essential oil against *S. aureus* depend on the bacterial strain and essential oil composition [9,12,14,15]. In silico molecular docking by simulating the mechanism of ATPase inhibitory activity through aligned structures of the catalytic domains of histidine kinase KdpD from *S. aureus* shows that neryl acetate and *α*-selinene are promising antimicrobial agents against this bacterium [9]. On the other side, strong negative correlations are noted between the content of 2-methylbutyl angelate, caryophyllene oxide, and *ar*-curcumene in the oils and their MIC values toward *S. aureus* [14]. Based on these studies, it can be assumed that there is an additive and synergistic effect among the *H. italicum* essential oil compounds on the antimicrobial potential for *S. aureus*. Therefore, it could be said that the antimicrobial efficacy of *H. italicum* essential oil against *S. aureus* varies significantly across different strains and compositions, as indicated by inhibition zone diameter, MIC, and MBC values. This variability underscores the importance of the chemical composition of the essential oil in determining its antimicrobial potency.

#### 3.1.2. *Staphylococcus epidermidis*

*Staphylococcus epidermidis* is a significant member of the human skin microbiota but is also a formidable opportunistic pathogen, especially in healthcare environments. It frequently causes infections related to medical devices due to its ability to form biofilms. These infections, including bloodstream infections and endocarditis, can be particularly difficult to treat. The issue of antimicrobial resistance in *S. epidermidis* is becoming increasingly critical. Methicillin-resistant *S. epidermidis* (MRSE) is particularly problematic due to resistance genes like the staphylococcal cassette chromosome mec (SCCmec), which confers resistance to methicillin and other antibiotics. Research has shown that MRSE is commonly found on the hands of hospital workers and healthy individuals alike, highlighting its widespread nature [31]. Additionally, strains of S. epidermidis isolated from patients often exhibit multidrug resistance, posing significant treatment challenges [32].

*H. italicum* essential oil has demonstrated substantial antimicrobial activity against various bacterial strains, including *S. epidermidis.* This suggests its potential as an effective alternative or adjunctive therapy for infections caused by antibiotic-resistant strains, particularly in scenarios where biofilms are involved. Only five studies investigated the antimicrobial activity of *H. italicum* essential oil against *S. epidermidis* (Table 3). The antimicrobial activity of the essential oil against *S. epidermidis* has been analyzed in various studies, demonstrating significant variability in its effectiveness.

Aćimović et al. [13] reported a Minimal Inhibitory Concentration (MIC) and Minimal Bactericidal Concentration (MBC) of 454.5 µg/mL for strain ATCC 12228. Genčić et al. [14] observed MIC values ranging from 0.60 to 2.50 mg/mL for the same strain, depending on the essential oil sample. Djihane et al. [21] recorded an inhibition zone diameter of 23.0 mm with both MIC and MBC at 25.3 µg/mL concentrations. Chinou et al. [28] determined a MIC value of 3.25 mg/mL for ATCC 12228. For clinical isolates, Malenica Staver et al. [19] found an inhibition zone diameter of 7.5 mm, with MIC and MBC values of 6.4 mg/mL, while for methicillin-resistant strain (MRSE), they reported an inhibition zone diameter of 8.0 mm with MIC and MBC values of 12.8 mg/mL. These data underscore the variability in the antimicrobial effectiveness of *H. italicum* essential oil against different strains of *S. epidermidis*, highlighting its potential but also the need for standardization in essential oil sample testing. Compared to *S. aureus*, *S. epidermidis* (ATCC 12228 and MRSA) is similar to *H. italicum* essential oil [13,14,19,21,28], while clinical isolates show a slightly smaller inhibition zone compared to *S. aureus* (7.5 vs. 8.5 mm, respectively) [19].

As can be seen from Table 3, inhibition zone diameters vary from 7.5 to 23.0 mm, while MIC/MBC values show significantly greater variation depending on the strain, the chemical composition of the essential oil, and the applied testing method. This is identical to *S. aureus*.

#### 3.1.3. *Bacillus cereus*

*Bacillus cereus* is widely recognized for its ability to cause foodborne illnesses, but it can also lead to severe infections, particularly in immunocompromised individuals. This bacterium is notable for its resistance to multiple antibiotics, thus complicating treatment. For example, *B. cereus* has been associated with serious conditions such as endocarditis, sepsis, and wound infections, which carry significant risks of morbidity and mortality [33]. Its resistance to many commonly used antibiotics, including beta-lactams, poses challenges in clinical management [34]. *B. cereus* exhibits significant antimicrobial resistance, complicating treatment protocols. This bacterium often shows resistance to beta-lactams, such as penicillins and cephalosporins, and even some carbapenems, making infections difficult to treat effectively [35]. Its ability to acquire resistance genes exacerbates the issue, leading to persistent and recurrent infections despite intensive antimicrobial therapy [36].

The increasing resistance of *B. cereus* to conventional antibiotics has prompted the investigation of alternative antimicrobial agents, such as essential oils. *H. italicum* essential oil has demonstrated significant antimicrobial properties against various pathogens, including *B. cereus.* A total of six papers referred to the antimicrobial activity of *H. italicum* essential oil against *B. cereus*, and the results are listed in Table 4. In the study Zeremski et al. [9], *B. cereus* responded to three *H. italicum* essential oil samples. Still, an antimicrobial effect was absent for the sample with the lowest α-pinene content (9.75%) and the highest content of total ketones (7.50%) and total esters (12.42%). For other samples, inhibition zone diameters ranged between 7.33 and 12.67 mm for *B. cereus* ATCC 11778, while for *B. cereus* ATCC 14579, Servi and Servi [10] reported a 13.00 mm inhibition zone. Genčić et al. [14] for *B. cereus* ATCC 11778 reported MIC between 1.2 and 10.0 mg/mL depending on the chemical composition of *H. italicum* essential oil, while Aćimović et al. [13], for the same strain, reported a MIC/MBC greater than 454.50 μL/mL. Additionally, Dzamic et al., [17] for human isolate *B. cereus*, noted a MIC of 2.5 mg/mL and an MBC > 2.5 mg/mL. Similarly, Djihane et al. [21] achieved a significantly lower MIC than MBC (3.162 μL/mL and 12.65 μL/mL, respectively).

#### 3.1.4. *Bacillus subtilis*

*Bacillus subtilis* is a widely studied, Gram-positive bacterium known for its role in soil nutrient cycling and its use in biotechnology and industry. Beyond its industrial and environmental significance, *B. subtilis* has notable impacts on human health, particularly through its probiotic properties. However, it is also important to recognize its potential for antimicrobial resistance, which can complicate its use and management in various applications. The bacterium has been shown to enhance the immune system, making it beneficial as a probiotic supplement [37]. However, studies on the antimicrobial resistance of Bacillus species, including *B. subtilis*, highlight the necessity to carefully consider its resistance patterns when used in clinical or agricultural settings. The emergence of antimicrobial resistance in *Bacillus subtilis*, though less common compared to more notorious pathogens, poses a significant challenge. The bacterium exhibited resistance to several antibiotics, which is particularly concerning in clinical environments, where it may act as an opportunistic pathogen [35].

A total of five papers referred to the antimicrobial activity of *H. italicum* essential oil against *B. subtilis*, and the results are listed in Table 5. Studies have shown that this essential oil can effectively inhibit the growth of several bacterial strains, suggesting its potential as a complementary treatment option for infections caused by antibiotic-resistant bacteria. Integrating *H. italicum* essential oil into treatment regimens could provide a valuable alternative, especially in cases where conventional antibiotics fail. In *H. italicum* essential oil, suppressed *B. subtilis* growth in inhibition zone diameters of from 8.00 to 12.00 mm depends on the bacterial strain (ATCC 19659 and ATCC 6633) [10,15] and essential oil composition [15]. MIC value ranged from 1.4 μg/mL [18] to 10.00 μg/mL [10] to 12.65 μg/mL [21], while the MBC for *B. subtilis* ATCC 6633 was the same as the MIC (1.4 μg/mL) [18], but the MBC for ATCC 9372 was significantly higher (50.6 μg/mL) [21]. Cui et al. [24] express MIC and MBC values in percentages: 0.05 and 0.1%, respectively.

Apart from these two species, *B. pumilus* and *B. spizizenii* were also studied by Cui et al. [24] and Aćimović et al. [13] (results are given in Section 3.1.7).

#### 3.1.5. *Listeria monocytogenes*

*Listeria monocytogenes*, a pathogen known for causing listeriosis, poses significant risks, particularly to pregnant women, neonates, the elderly, and immunocompromised individuals. This bacterium can lead to severe outcomes, such as sepsis, meningitis, and neonatal infections. The primary treatment for listeriosis typically involves ampicillin combined with gentamicin, though resistance to multiple antibiotics has been observed in various strains [38]. This bacterium demonstrates varying degrees of resistance to several antibiotics, complicating treatment options. Resistance to commonly used antibiotics, such as trimethoprim–sulfamethoxazole, tetracycline, and gentamicin, has been reported [39]. Additionally, in some regions, strains have shown resistance to penicillin G and amoxicillin, which are critical in the treatment regimen for listeriosis [40]. Continuous monitoring and management of antimicrobial resistance are essential to ensure effective treatment.

The antimicrobial properties of *H. italicum* essential oil present a promising alternative for managing infections caused by resistant strains of *L. monocytogenes*, emphasizing the need for ongoing research and development of alternative antimicrobial therapies. Zeremski et al. [9] showed that the inhibition zone diameter of *H. italicum* essential oil on *L. monocytogenes* varied between 7.33 and 17.00 mm depending on the essential oil’s chemical composition. The most potent compounds in *H. italicum* essential oil are α-selinene and neryl acetate. Investigations dealing with MIC/MBC showed almost similar values: 454.50/454.50 μg/mL [13], >15.00/>15.00 mg/mL [17], and <1.4/1.4 μg/mL [18]. Meanwhile Djihane et al. [21] did not detect antimicrobial activity. All of these are summarized in Table 6.

Apart from *L. monocytogenes*, two more strains were used in investigations of *H. italicum* essential oil antimicrobial activity: *L. innocua* and *L. ivanovii* [13] (results are given in Section 3.1.7).

#### 3.1.6. *Enterococcus faecalis*

*Enterococcus faecalis* is a significant pathogen known for its role in causing a variety of infections, including urinary tract infections, endocarditis, and bacteremia. This bacterium is particularly concerning in hospital settings due to its ability to survive harsh conditions and its high levels of antibiotic resistance. Infections caused by *E. faecalis* can be challenging to treat, particularly because this organism has developed resistance to many commonly used antibiotics [41]. Four papers investigated the potential of *H. italicum* essential oil against E. *faecalis*, and the results are summarized in Table 7. It can be concluded from the table that different strains show different sensitivity to *H. italicum* essential oil. In addition, variations also occur depending on the chemical composition of the essential oil.

The antimicrobial activity against *E. faecalis* exhibits significant variability across different strains and studies. For instance, Aćimović et al. [13] found that the tested concentration of essential oil (454.50 μg/mL) did not have inhibited effects against the referent strain, ATCC 29212. Conversely, Genčić et al. [14] reported MIC values ranging from 2.50 to 10.00 mg/mL for strain ATCC 19433; however, no MBC was detected, indicating variable efficacy dependent on the essential oil sample. Notably, Šćepanović et al. [18] demonstrated strong antimicrobial activity against the same strain (ATCC 19433), with low MIC and MBC values of 7.1 μg/mL and 14.0 μg/mL, respectively. Djihane et al. [21] found moderate effectiveness of the essential oil against strain ATCC 49452, with MIC and MBC values of 50.6 μg/mL and 101.2 μg/mL. These findings highlight the essential oil’s potential as an antimicrobial agent, while also underscoring the importance of strain-specific testing and consideration of sample variability.

Apart from *E. faecalis*, *E. cereus* was investigated for *H. italicum* essential oil antimicrobial activity [21] (results are given in Section 3.1.7).

#### 3.1.7. Other Gram-Positive Bacteria

A total of 11 Gram-positive bacteria were studied only once (details given in Table 8). *H. italicum* essential oil showed a MIC value of 0.375 mg/mL, indicating good antimicrobial activity against *Streptococcus pneumoniae* [11], which is a major cause of pneumonia, meningitis, and sepsis, with a high level of resistance to penicillin and erythromycin. *Listeria innocua* and *Listeria ivanovii*, although less commonly pathogenic than *L. monocytogenes*, can still cause severe infections, particularly in immunocompromised individuals. The antimicrobial potential of *H. italicum* essential oil is not defined since the obtained values of MIC were above the tested concentration [13]. Given the rising concern over antibiotic resistance in *Bacillus* species, alternative antimicrobial agents, such as essential oils, are being investigated. Except for *B. cereus* and *B. subtilis*, *H. italicum* essential oil has demonstrated different antimicrobial activity against other strains, such as *B. pumilis* and *B. spizizenii* [13,24]. Namely, as an environmental bacterium that occasionally causes human infections, including sepsis, endocarditis, and food poisoning, *B. pumilis* was sensitive to the effect of the tested oil [24].

On the other hand, some isolates have been found to be resistant to various antibiotics, except quinolones and vancomycin, posing a challenge in clinical management [42], so the gained result is very promising for alternative strategies against this *Bacillus* specie. Additionally, *B. pumilus* has shown resistance to several antibiotics, complicating clinical treatment protocols. A study revealed that *B. pumilus* isolates from bloodstream infections exhibited resistance to all antibiotics tested, except quinolones and vancomycin [43]. On the other hand, *B. spizizenii*, a close relative of *B. subtilis*, shares similar characteristics in terms of environmental resilience and potential industrial applications. The obtained resistance in the work of Aćimović et al. [13] was expected; however, its role as a pathogen and its antimicrobial resistance profile are less well-documented. This species can serve as a model organism for studying spore formation and bacterial genetics due to its close genetic relationship with *B. subtilis*. Both are notable for their environmental ubiquity and potential to cause infections, especially in immunocompromised individuals. The increasing antimicrobial resistance observed in these species necessitates the exploration of alternative treatments. There are very specific and unique data indicating that *H. italicum* essential oil offers a potential alternative for these two species, highlighting the need for further research into its efficacy and application in clinical settings.

As can be seen in Table 8, the essential oil showed moderate activity, with MIC and MBC values of 454.50 μg/mL against *Rhodococcus equi* (causes respiratory infections in humans and animals, particularly in immunocompromised hosts. [13]. *Sarcina lutea* is generally sensitive bacterium but can develop resistance. The essential oil’s MIC values ranged from 2.5 to 10.0 mg/mL, indicating variable effectiveness in view of tested oil against the referent strain [14]. Highly resistant to many standard antibiotics, *Mycobacterium avium* requires complex treatment regimens, while Peruč et al. [20] proved that essential oil had good activity, with a MIC and MBC of 3.2 mg/mL. *Mycobacterium intracellulare*, similar to *M. avium*, causes pulmonary infections in immunocompromised individuals, but the essential oil showed similar efficacy with a MIC and MBC of 3.2 mg/mL [20]. The essential oil showed a very low MIC of 0.79 μg/mL for *E. cereus*, indicating strong antimicrobial activity against this bacterium, but the same oil in higher concentration (MIC of 6.325 μg/mL and MBC of 12.65 μg/m) had to be used against *Micrococcus luteus* [21].

### 3.2. Gram-Negative Bacteria

#### 3.2.1. *Escherichia coli*

*Escherichia coli* remains a major public health concern due to its pathogenicity and significant levels of antimicrobial resistance. Antimicrobial resistance in *E. coli* varies widely across different regions and clinical settings. For instance, a study conducted in Germany highlighted a substantial rise in antibiotic resistance among *E. coli* isolates during antibiotic therapy, with resistance levels doubling or more during treatment and returning to baseline levels only after the cessation of therapy [44]. Another study demonstrated that *E. coli* isolates from urinary tract infections in inpatients showed high levels of multidrug resistance (76.5%), with significant resistance to commonly used antibiotics, such as ampicillin (88.4%), amoxicillin–clavulanic acid (74.4%), and ceftriaxone (71.4%) [45]. Additionally, a longitudinal study in Tanzania found that resistance to ampicillin and trimethoprim/sulfamethoxazole was highly prevalent among *E. coli* isolates from young children, with pathogenic strains showing higher resistance rates compared to non-pathogenic strains [46]. Given the increasing resistance of *E. coli* to conventional antibiotics, alternative treatments, such as essential oils, are being explored. *H. italicum* essential oil has demonstrated significant antimicrobial activity against various bacterial strains, including antibiotic-resistant *E. coli*. As mentioned above, a total of 15 studies examined the antimicrobial activity of *H. italicum* essential oil on *E. coli* (Table 9). A total of five studies indicates that there is no antimicrobial activity [9,10,18,21,28], while the results from eight papers show that the MIC/MBC, depending on the bacterial strains and the chemical composition of the essential oil [12,13,14,16,17,19,24,26]. Additionally, the inhibition zone varied from 6.00 to 66.00 mm [15,19,22,26]. Research indicates that this essential oil can effectively inhibit the growth of resistant bacterial strains, suggesting its potential as a complementary or alternative treatment option. For instance, studies have shown that natural compounds, like essential oils, can target bacterial cell walls and disrupt biofilm formation, which are critical for bacterial survival and resistance [47].

Such uneven results are certainly a consequence of the chemical composition of the essential oil. Studies by Weglarz et al. [12] and Genčić et al. [14] indicate this. The first study [12] investigated essential oil obtained from inflorescences rich in neryl acetate and nerol and herbs with dominant neryl acetate and *α*-pinene. The second study [14] dealing with four commercial essential oils with neryl acetate, *α*-pinene, *γ*-curcumene, and *ar*-curcumene in different proportions. In conclusion, the authors stated that the antimicrobial activity of *H. italicum* essential oil is a result of synergistic action between main constituents, as well as the presence of some minor compounds.

*H. italicum* essential oil has shown varying inhibition zone diameters against *E. coli* strains, reflecting its antimicrobial potency. A study conducted by Aćimović et al. [13], with two *E. coli* strains (ATCC 8739 and ATCC 10536), showed the same MIC value for both, >0.4545 mg/mL. Similarly, Malenica Staver et al. [19] reported MIC/MBC for *E. coli* ATCC 25922 and extended spectrum beta-lactamase strain (ESBL+) of 12.80 mg/mL, while the inhibition zone was 6.00 mm. On the other hand, the sensitivity of E. coli ATCC 25922 strongly depends on the chemical composition of *H. italicum* essential-oil composition.

Oliva et al. [16] and Mastelić et al. [26], in an investigation, used *E. coli* ATCC 25922 and observed MIC values > 5.00 and 7.00 mg/mL, respectively, while Dzamic et al. [17], for *E. coli* ATCC 35210, reported MIC 2.50 mg/mL. Genčić et al. [14] observed a MIC range of 2.50–10.00 µg/mL depending on the essential-oil composition. Better results showed essential-oil samples rich in sesquiterpenes (50.9–55.2%), in comparison to samples with a higher share of monoterpenes (47.2–55.0%). These authors hypothesize that the antimicrobial activity of *H. italicum* essential oil depends primarily on the chemical composition of the sample, as well as on the method used for MIC evaluation. Weglarz et al. [12] reported a MIC ranging from 32.00 mg/mL (for herb essential oil) to 64.00 mg/mL (for inflorescence essential oil). Herb essential oil, rich in neryl acetate (20.3%) and α-pinene (10.4%), expressed better activity in comparison to inflorescence oil with 16.4% neryl-acetate and 15.7% nerol. However, MBC was the same for both essential oil samples, >64.00 mg/mL.

The inhibition zone diameters (mm) of *H. italicum* essential oil against *E. coli* were investigated by Mollova et al. [15], Bouchaala et al. [22], and Mastelić et al. [26]. The highest resilience was showing *E. coli* ATCC 8739 (8.00 mm), and essential-oil compositions have no influence on these values [15]. The zone diameter of 66.00 mm observed in the study by Mastelic [26] indicates an exceptionally high antimicrobial activity, significantly surpassing the 14.00 mm zone diameter reported by Bouchaala et al. [22]. The reason for this high difference of inhibition zone diameter can be in different main constitutes defined by two research groups. Namely, Bouchaala et al. [22] reported isopropyl tetradecanoate (12.1%) and α-pinene (12.0%) as main compounds, while Mastelić et al. [26] investigated samples rich in neryl acetate (11.5%) and α-pinene (10.2%). However, in their research, Mollova et al. [15] included two samples, one from Bosnia and one from France, with the same dominant compounds (neryl acetate and γ-curcumene) but without differences in antimicrobial activity.

#### 3.2.2. *Pseudomonas aeruginosa*

*Pseudomonas aeruginosa* remains a significant threat in healthcare settings due to its high levels of antimicrobial resistance and ability to cause severe infections. The promising antimicrobial properties of essential oils offer a potential alternative for treating infections caused by resistant strains of this bacterium. It is very important to highlight that *P. aeruginosa* is notorious for its high level of resistance to multiple antibiotic classes, thus complicating many clinical treatments. Studies have shown that this pathogen frequently exhibits resistance to beta-lactams, aminoglycosides, and fluoroquinolones. In brief, a study in the United States indicated that the prevalence of multidrug-resistant *P. aeruginosa* increased from 15.4% in 1999 to 26% in 2012, highlighting the growing challenge of treating infections caused by this pathogen [48].

Essential oils, such as *H. italicum* essential oil, have shown promising antimicrobial properties. Research has demonstrated that *H. italicum* essential oil can effectively inhibit the growth of resistant bacterial strains, making it a potential complementary treatment option. The essential oil’s antimicrobial activity is attributed to its ability to disrupt bacterial cell walls and inhibit biofilm formation, critical factors for bacterial survival and resistance [49]. Specific studies have highlighted the efficacy of *H. italicum* essential oil against *P. aeruginosa*, suggesting its role as a natural alternative in managing infections caused by resistant strains. A total of 14 papers referred to the antimicrobial activity of *H. italicum* essential oil against *P. aeruginosa*, and they are listed in Table 10. A detailed analysis shows that the MIC/MBC values express a wide range of variations [10,11,12,13,16,17,19,21,24,28], as well as the inhibition zone diameters (from 6.00 to 66.00 mm) [10,15,19,26]. On the other side, Zeremski et al. [9] and Šćepanović et al. [18] did not detect the antimicrobial activity of *H. italicum* essential oil against *P. aeruginosa*.

*H. italicum* essential oil has shown varying inhibition zone diameters against *P. aeruginosa* strains, ranging from 6.00 to 66.00 mm for ATCC 25923 and ATCC 9027 [10,15,19,26]. Servi and Servi [10] observed a 10 mm inhibition zone for the essential oil rich in neryl acetate (15.6%), α-pinene (11.5%), and γ-curcumene (11.3%) in the case of *P. aeruginosa* ATCC 27853. Malenica Staver et al. [19] observed inhibition zones of 6.00 mm for the same strain in the case of oil with dominant α-pinene (21.6%) and γ-curcumene (21.6%). The highest inhibition zone of 66.00 mm was reported by Mastelić et al. [26] for essential oil rich in neryl acetate (11.5%) and α-pinene (10.2%), while Mollova et al. [15] reported a zone of 8 mm for ATCC 9027 for the two essential oils obtained from of *H. italicum* grown in Bulgaria (Bosnia and France origin). The MIC/MBC, expressed in µg/mL, for *P. aeruginosa* ranged from >0.4545 to 12.56 [10,13,21]. Moreover, MIC/MBC expressed in mg/mL ranged from 0.625 mg/mL [11] to 1.5 mg/mL [17] to 3.75 mg/mL [28] to 12.80 mg/mL [19], while Weglarz et al. [12] states a significantly higher concentration from 32.00 to 64.00 mg/mL depending on the chemical composition of the essential oil (herb or inflorescence). The antimicrobial efficacy of *H. italicum* essential oil against *P. aeruginosa* varies significantly across different strains (ATCC 27853, ATCC 9027, or CRPA) and main compounds from essential oil, as indicated by inhibition zone diameter, MIC, and MBC values.

#### 3.2.3. *Salmonella enterica*

*Salmonella enterica* is a significant cause of foodborne illness, and its antimicrobial resistance presents a major public health challenge. Three different strains of serovar Typhimurium (ATCC 13311, ATCC 14028, and ATCC 19659) were studied [9,13,17,24], as well as only one serovar Enteritidis (ATCC 13076) [13,14,18] (Table 11). The antimicrobial effectiveness of *H. italicum* essential oil against *Salmonella enterica* varies significantly depending on the strain and study. The most promising results were seen in Cui et al. [24], where low concentrations effectively inhibited ATCC 19659. In contrast, other studies, such as that by Aćimović et al. [13], reported that MIC and MBC values are above 454.50 µg/mL, indicating lower effectiveness. Variability in results suggests a need for standardized testing methods and further investigation into the factors influencing efficacy.

Regarding MIC values, there was considerable variation among the studies (Table 11). Aćimović et al. [13] reported MIC values greater than 454.50 µg/mL for strains ATCC 13076 and ATCC 14028, indicating a relatively low antimicrobial effectiveness at the tested concentrations. Genčić et al. [14] found a broader MIC range of 5.0–10.0 mg/mL for ATCC 13076, suggesting variability depending on the essential oil sample used. Dzamic et al. [17] reported a MIC of 2.5 mg/mL for strain ATCC 13311, indicating moderate effectiveness. In contrast, Cui et al. [24] reported a significantly lower MIC of 0.05% for ATCC 19659, suggesting high effectiveness at relatively low concentrations. On the other hand, Aćimović et al. [13] found MBC values greater than 454.50 µg/mL for both ATCC 13076 and ATCC 14028, aligning with their MIC findings and indicating limited bactericidal activity at these concentrations. Dzamic et al. [17] reported an MBC of 2.5 mg/mL for ATCC 13311, reflecting a moderate level of effectiveness. Cui et al. [24] again demonstrated higher efficacy, with an MBC of 0.10% for ATCC 19659. No MBC data were provided by Genčić et al. [14] for ATCC 13076 or by Šćepanović et al. [18] for ATCC 13076. The antimicrobial effectiveness of the essential oil against *Salmonella enterica* varies significantly across different strains and studies. The most promising results were observed in the study by Cui et al. [24], where low concentrations of the essential oil effectively inhibited and killed the ATCC 19659 strain. In contrast, other studies, such as those by Aćimović et al. [13], reported that MIC and MBC values are higher than 454.50 µg/mL, indicating lower effectiveness. This variability highlights the need for standardized testing methods and further investigation into the factors influencing the essential oil’s antimicrobial efficacy.

#### 3.2.4. *Klebsiella pneumoniae*

*Klebsiella pneumoniae* is a major pathogen associated with hospital-acquired infections, posing significant challenges due to its increasing resistance to antibiotics. A total of five papers referred to the antimicrobial activity of *H. italicum* essential oil against *K. pneumoniae* (Table 12). The antimicrobial activity of *H. italicum* essential oil against various strains of *K. pneumoniae* was evaluated across several studies. Oliva et al. [16] tested the essential oil on a carbapenem-resistant *K. pneumoniae* (CR-Kp) isolate and reported a Minimal Inhibitory Concentration (MIC) and Minimal Bactericidal Concentration (MBC) both greater than 5.0%, indicating limited effectiveness at the concentrations tested. In contrast, Djihane et al. [21] did not detect any inhibitory or bactericidal activity against strain ATCC 4352.

Bouchaala et al. [22] observed an inhibition zone diameter of 13.00 mm for strain ATCC 700603; however, the MIC and MBC values were not reported. This suggests moderate antimicrobial activity based on the inhibition zone. Cui et al. [24] found a MIC of 0.05% and an MBC of 0.10% for strain ATCC 13883, demonstrating significant antimicrobial effectiveness at low concentrations. Similarly, Chinou et al. [28] reported a MIC of 3.50 mg/mL for the same strain, though MBC was not determined. The studies reviewed present a varied picture of the antimicrobial effectiveness of *H. italicum* essential oil against *Klebsiella pneumoniae.* Inhibition zone diameters were reported only by Bouchaala et al. [22], showing a moderate inhibition of 13.00 mm for strain ATCC 700603. MIC values ranged significantly, with Oliva et al. [16] reporting high values (>5.0%) for a carbapenem-resistant isolate, indicating lower effectiveness, while Cui et al. [24] reported much lower values (0.05%) for ATCC 13883, suggesting high efficacy. Chinou et al. [28] found an intermediate MIC of 3.50 mg/mL for the same strain. MBC values also varied, with Oliva et al. [16] reporting an MBC greater than 5.0% and Cui et al. [24] reporting a much lower MBC of 0.10%.

*K. aerogenes* was also investigated in one study [13] (results are given in Section 3.2.7).

#### 3.2.5. *Acinetobacter baumannii*

*Acinetobacter baumannii* is a significant nosocomial pathogen known for its ability to develop resistance to multiple antibiotics, posing severe challenges in clinical settings. Exploring alternative antimicrobial agents, such as essential oils from plants like *H. italicum*, is essential for addressing infections caused by multidrug-resistant (MDR) strains [16,50,51]. A total of three papers referred to the antimicrobial activity of *H. italicum* essential oil against *A. baumannii* (Table 13).

Genčić et al. [14] evaluated the essential oil against strain ATCC 19606, reporting Minimal Inhibitory Concentration (MIC) values ranging from 1.2 to 5.0 µg/mL, depending on the essential oil sample used. However, the Minimal Bactericidal Concentration (MBC) values were not determined in this study. Oliva et al. [16] examined the essential oil’s effectiveness against a carbapenem-resistant *A. baumannii* (CR-Ab) isolate. The study reported MIC and MBC values at 5%, indicating limited effectiveness at the tested concentrations. Malenica Staver et al. [19] provided a more comprehensive analysis, testing the essential oil against two strains: ATCC BAA-1605 and ATCC 19606. For ATCC BAA-1605, the inhibition zone diameter was 14.0 mm, with MIC and MBC values both at 3.2 mg/mL. Similarly, for ATCC 19606, the inhibition zone diameter was 13.5 mm, with the same MIC and MBC values of 3.2 mg/mL. In summary, Genčić et al. [14] reported relatively low MIC values (1.2–5.0 µg/mL) for ATCC 19606, suggesting significant antimicrobial activity. Conversely, Oliva et al. [16] reported higher MIC and MBC values (5%) for a carbapenem-resistant isolate, indicating lower effectiveness at the concentrations tested. Malenica Staver et al. [19] reported moderate inhibition zone diameters (13.5–14.0 mm) and consistent MIC and MBC values (3.2 mg/mL) for both tested strains, highlighting moderate antimicrobial effectiveness. Some studies, like those by Genčić et al. [14], indicate significant effectiveness at lower concentrations, while others, such as that of Oliva et al. [16], report limited efficacy against carbapenem-resistant strains. These findings underscore the need for standardized testing methods and further research to explore the potential of *H. italicum* essential oil as an alternative antimicrobial agent.

#### 3.2.6. *Proteus mirabilis*

A total of two papers referred to the antimicrobial activity of *H. italicum* essential oil against *Proteus mirabilis* (Table 14), a well-known Gram-negative bacterium commonly associated with urinary tract infections (UTIs) and known for its ability to form biofilms and develop resistance to multiple antibiotics. In a study by Šćepanović et al. [18], the essential oil was tested against the strain ATCC 25933. However, the study did not report inhibition zone diameters, while MIC or MBC values are not defined, indicating that the specific antimicrobial activity was not detected. Conversely, Djihane et al. [21] provided a more detailed analysis by testing the essential oil against strain ATCC 35659. This study reported a MIC value of 1.581 µg/mL and an MBC value of 3.162 µg/mL, demonstrating antimicrobial potential against this strain.

*Proteus hauseri* was also investigated in one study [13] (results are given in Section 3.2.7).

#### 3.2.7. Other Gram-Negative Bacteria

A total of nine Gram-negative bacteria were studied only once in view of the antimicrobial potential of the targeted essential oil. Table 15 presents a comprehensive analysis of the antimicrobial properties of *H. italicum* essential oil against a range of Gram-negative bacteria. The results indicate variability in the essential oil’s efficacy, which is strain-dependent. This variability is critical for understanding its potential application in treating bacterial infections. The essential oil exhibited significant antimicrobial activity against *Haemophilus influenzae* DSM 4690 and *Haemophilus parainfluenzae* DSM 8978, with both strains showing MIC values of 0.312 mg/mL [11]. This indicates a strong inhibitory effect, suggesting that the essential oil can effectively impede the growth of these bacteria at relatively low concentrations. Similarly, *Streptococcus pneumoniae* DSM 20566 demonstrated moderate sensitivity to the essential oil, with a MIC value of 0.375 mg/mL [11], reinforcing the potential of *H. italicum* as an antimicrobial agent against respiratory pathogens. In contrast, *Klebsiella aerogenes* ATCC 13048 and *Proteus hauseri* ATCC 13315 were notably resistant, with both strains having MIC and MBC values exceeding 454.50 µg/mL [13]. This high resistance level highlights a significant limitation in the essential oil’s efficacy against these particular strains, indicating that much higher concentrations are needed to achieve bactericidal effects, which may not be practical for therapeutic use.

*Enterobacter aerogenes* ATCC 13048 presented a more varied response, with MIC values ranging between 2.5 and 5.0 mg/mL, depending on the essential oil sample used [14]. This variability underscores the importance of standardizing essential oil samples for consistent antimicrobial testing and suggests that different batches of the oil can have markedly different levels of effectiveness. *Pseudomonas syringae* and *Shigella* sp. were assessed using inhibition zone diameters, which were 9 mm and 13 mm, respectively [22]. Although MIC and MBC values were not provided for these strains, the inhibition zones suggest a measurable degree of antimicrobial activity. The larger inhibition zone for *Shigella* sp. implies a more potent effect compared to *Pseudomonas syringae*, but further quantitative data would be necessary to draw more definitive conclusions. Finally, *Enterobacter cloacae* ATCC 13047 demonstrated moderate-to-low susceptibility, with a MIC value of 3.5 mg/mL [28]. This finding aligns with the broader trend observed for Gram-negative bacteria, where susceptibility to the essential oil varies widely and tends to be strain-specific. The essential oil of *H. italicum* exhibits significant antimicrobial activity against certain Gram-negative bacteria, particularly *Haemophilus influenzae*, *Haemophilus parainfluenzae*, and *Streptococcus pneumoniae*. However, its effectiveness is markedly reduced against strains such as *Klebsiella aerogenes* and *Proteus hauseri*.

### 3.3. Yeast

#### 3.3.1. *Candida albicans*

*Candida albicans* is a common pathogenic yeast responsible for various human infections, particularly in immunocompromised individuals. It is part of the normal microbiota of the oral cavity, gastrointestinal tract, and vagina, but under certain conditions, it can overgrow and cause diseases such as thrush, vulvovaginal candidiasis, and invasive candidiasis [52]. The pathogenicity of *C. albicans* is attributed to its ability to switch between yeast and hyphal forms, form biofilms, and produce a variety of virulence factors. The rise of antifungal resistance in *C. albicans* highlights the need for alternative treatments, such as plant-derived essential oils, which have shown promising antifungal properties [53]. The antifungal activity of *H. italicum* essential oil against *C. albicans* has been extensively studied, with varied results due to differences in the chemical composition of the essential oils used, the strains of *C. albicans* tested, and the methodologies employed in the studies. A total of ten studies were dealing with the antifungal activity of *H. italicum* essential oil against *C. albicans* (Table 16). The inhibition zone diameters, which measure the extent to which *H. italicum* essential oil can inhibit the growth of *C. albicans*, varied significantly across the studies.

Zeremski et al. [9] reported inhibition zones ranging from 7.67 to 13.67 mm for the ATCC 10231 strain, indicating a substantial variation that could be due to different compositions of the essential oil samples tested. Similarly, Mollova et al. [15] found an inhibition zone of 8.00 mm for the same strain, while Mastelić et al. [26] observed an inhibition zone of 9.00 mm. In contrast, Malenica Staver et al. [19] noted a smaller inhibition zone of 7.5 mm for an ESBL+ clinical isolate, suggesting that clinical isolates may respond differently compared to standard laboratory strains. The MIC values, which indicate the lowest concentration of essential oil required to inhibit visible growth of *C. albicans*, also displayed significant variation. Genčić et al. [14] reported MIC values between 2.50 and 5.00 mg/mL for the ATCC 10231 strain. Dzamic et al. [17] documented a considerably higher MIC of 24.17 mg/mL for the same strain, which could reflect differences in the chemical composition of the essential oil or variations in testing conditions. Malenica Staver et al. [19] found a MIC of 6.4 mg/mL for an ESBL+ clinical isolate, suggesting a higher tolerance compared to standard strains. Djihane et al. [21] reported a MIC of 6.325 μg/mL, demonstrating significant efficacy at a lower concentration, while Mastelić et al. [26] indicated a MIC of 5.00 μL/mL. Oliva et al. [16] identified a MIC of 5.00% for the ATCC 14053 strain, further highlighting the variability in effectiveness based on oil composition and test conditions. The MFC values, representing the lowest concentration required to kill *C. albicans*, also varied. Dzamic et al. [17] reported an MFC of 24.17 mg/mL for ATCC 10231, while Djihane et al. [21] found an MFC of 12.65 μg/mL for the same strain, indicating a high fungicidal activity at lower concentrations. Oliva et al. [16] reported an MFC of 5.00% for ATCC 14053.

Two studies, Šćepanović et al. [18] and Bouchaala et al. [22], reported that *C. albicans* exhibited no sensitivity to *H. italicum* essential oil, with no detectable inhibition zones or MIC and MFC values. This lack of sensitivity could be due to specific strains used, differences in the oil composition, or variations in the testing methodologies. The antifungal activity of *H. italicum* essential oil against *C. albicans* demonstrates significant variability, which can be attributed to differences in essential oil composition, *C. albicans* strains, and testing methodologies. Despite this variability, the oil shows potential as an antifungal agent, though further standardization in testing methods is required to assess its efficacy accurately.

#### 3.3.2. *Saccharomyces cerevisiae*

*Saccharomyces cerevisiae* is a model organism extensively used in molecular and cellular biology research. It is crucial in various industrial fermentation processes, including breadmaking, brewing, and winemaking. Its significance extends to scientific research due to its well-characterized genetics and ease of manipulation. Studying the antifungal properties of substances like essential oils against S. cerevisiae is essential for both industrial applications and the development of new antifungal agents. The antimicrobial activity of H. italicum essential oil against S. cerevisiae has been investigated in several studies (Table 17), revealing variability influenced by the chemical composition of the essential oil, the strain tested, and the experimental methodologies.

Zeremski et al. [9] observed inhibition zone diameters ranging from 13.67 to 18.67 mm for the ATCC 9763 strain, indicating strong antifungal activity. In contrast, Mollova et al. [15] reported a smaller inhibition zone of 8.00 mm for the same strain, suggesting that differences in essential oil composition or experimental conditions significantly impact the results. Djihane et al. [21] provided Minimal Inhibitory Concentration (MIC) and Minimal Bactericidal Concentration (MBC) values, reporting a MIC of 6.325 μg/mL and an MBC of 12.65 μg/mL for the ATCC 9763 strain. These findings indicate that H. italicum essential oil can inhibit the growth of S. cerevisiae at low concentrations and achieve a bactericidal effect at slightly higher concentrations. The observed variability in inhibition zones and concentration values underscores the importance of standardizing essential oil compositions and testing methodologies to assess antifungal efficacy accurately.

### 3.4. Fungi

#### 3.4.1. *Aspergillus* Species

The antifungal properties of *H. italicum* essential oil against various *Aspergillus* species have been evaluated across several studies (Table 18), revealing a spectrum of efficacy depending on the species, strain origin, and essential oil composition. Aspergillus is a genus of fungi that is highly relevant in antimicrobial testing due to its widespread presence and significant impact on human health, agriculture, and various industries. There is growing evidence of antifungal resistance among *Aspergillus* species, which complicates treatment protocols. The development of resistance to commonly used antifungal agents, such as azoles, necessitates the search for new and effective antifungal compounds [54]. Testing the efficacy of alternative treatments, including essential oils like *H. italicum*, against *Aspergillus* is crucial in the fight against resistant strains.

According to Table 18, Zeremski et al. [9] investigated the effect of *H. italicum* essential oil on *Aspergillus brasiliensis* (ATCC 16404) but did not detect inhibition zone diameter, Minimal Inhibitory Concentration (MIC), or Minimal Bactericidal Concentration (MBC) values. In contrast, Mollova et al. [15] reported significant inhibition zones for *A. brasiliensis* (ATCC 16404), ranging from 17.41 to 18.00 mm; however, the MIC and MBC values were not determined in this study. Dzamic et al. [17] examined the essential oil’s effects on multiple Aspergillus species. For *Aspergillus fumigatus* (clinical isolate), *Aspergillus niger* (ATCC 6275), *Aspergillus ochraceus* (ATCC 12066), and *Aspergillus versicolor* (ATCC 11730), they found consistent MIC and MBC values of 14.50 mg/mL and 48.35 mg/mL, respectively. These results indicate a substantial inhibitory and bactericidal effect of the essential oil at relatively high concentrations. Additionally, Djihane et al. [21] reported lower MIC and MBC values for *A. niger* from their internal collection, with the MIC at 25.3 μg/mL and MBC at 50.6 μg/mL, suggesting a higher sensitivity of this strain to the essential oil. Stupar et al. [25] focused on culture heritage isolates from wood, specifically *A. niger* and *A. ochraceus*. They found MIC and MBC values of 75 μL/mL and 100 μL/mL, respectively, indicating that these isolates require lower concentrations of the essential oil for inhibition and bactericidal effects compared to other strains tested by Dzamic et al. [17]. This analysis shows that the efficacy of *H. italicum* essential oil against *Aspergillus* species varies considerably, influenced by strain origins and specific characteristics. For instance, the essential oil appears highly effective against *A. brasiliensis* in terms of inhibition zones but less so in terms of MIC and MBC values. Conversely, *A. niger* and *A. ochraceus* from different origins exhibit a broad range of sensitivity, with some strains requiring significantly higher essential oil concentrations for effective inhibition and killing. These findings underscore the need for standardized testing protocols and a deeper understanding of the essential oil’s chemical composition to assess its antifungal potential across diverse Aspergillus species accurately.

#### 3.4.2. *Penicillium* Species

The antimicrobial properties of *H. italicum* essential oil against various *Penicillium* species have been investigated, with varying degrees of efficacy depending on the specific species, strain origin, and essential oil composition, as for the previous fungal representative. A total number of four papers are summarized in Table 19. Zeremski et al. [9] studied the effect of *H. italicum* essential oil on *P. aurantiogriseum* ATCC 16025, but they did not detect any inhibition zone diameters, suggesting that this strain might be less sensitive to the essential oil. Conversely, Dzamic et al. [17] reported significant antimicrobial activity against *P. funiculosum* ATCC 36839 and *P. ochrochloron* ATCC 9112. Both species exhibited MIC values of 14.50 mg/mL and MBC values of 24.17 mg/mL, indicating that the essential oil can inhibit and kill these strains at relatively high concentrations. Stupar et al. [25] focused on a culture heritage isolate of *Penicillium* sp. from wood. They found MIC and MBC values of 25 μL/mL and 50 μL/mL, respectively. These lower values suggest that this particular isolate requires a lower concentration of essential oil for inhibition and bactericidal effects compared to the strains tested by Dzamic et al. [17].

The data indicate that *H. italicum* essential oil exhibits variable antimicrobial activity against different *Penicillium* species. The effectiveness appears to be species- and strain-specific, with some strains showing significant sensitivity to the oil, while others do not. This variability emphasizes the importance of further research to understand the factors influencing the antimicrobial efficacy of *H. italicum* essential oil, including its chemical composition and the characteristics of the target fungal strains.

#### 3.4.3. *Fusarium* Species

*Fusarium* species are significant in the context of antimicrobial testing due to their impact on human health, agriculture, and the emerging issue of antimicrobial resistance. *Fusarium* species are known to cause opportunistic infections in humans, especially in immunocompromised individuals, leading to conditions such as keratitis, onychomycosis, and disseminated infections. In agriculture, *Fusarium* species are notorious for causing substantial crop losses by infecting cereals, legumes, and other plants, leading to diseases such as *Fusarium* head blight and root rot [55]. These infections not only reduce crop yields but also contaminate food supplies with mycotoxins, posing severe health risks to humans and animals.

The data illustrated in Table 20 show that *H. italicum* essential oil exhibits variable antimicrobial activity against different *Fusarium* species. *F. solani* var. *coeruleum* appears to be highly sensitive to the essential oil, while *F. graminearum* and *F. oxysporum* var. *lycopersici* exhibit significant resistance, requiring substantially higher concentrations for effective inhibition. This variability underscores the importance of considering species-specific responses and the chemical composition of the essential oil when evaluating its antimicrobial efficacy.

Angioni et al. [27] evaluated the impact of *H. italicum* essential oil on *F. graminearum* and *Fusarium oxysporum* var. *lycopersici*. For *F. graminearum*, the Minimal Inhibitory Concentration (MIC) values ranged from 500 to 1000 mg/mL, indicating that relatively high concentrations of the essential oil are necessary to inhibit this species. In contrast, for *F. oxysporum* var. *lycopersici*, both MIC and Minimal Bactericidal Concentration (MBC) values exceeded 1000 mg/mL, suggesting significant resistance of this species to the essential oil. Mollova et al. [15] assessed the antimicrobial activity against *Fusarium moniliforme*, a clinical isolate, reporting an inhibition zone diameter of 8.00 mm. This result suggests moderate susceptibility of this strain to *H. italicum* essential oil, although MIC and MBC values were not provided. Djihane et al. [21] investigated *Fusarium solani* var. *coeruleum* from their internal collection, finding both MIC and MBC values to be 50.6 μg/mL. This indicates a high sensitivity of this *Fusarium* species to the essential oil, as lower concentrations were effective in both inhibiting and killing the fungus.

#### 3.4.4. *Trichoderma viride*

*Trichoderma* species are commonly found in soil and are known for their beneficial effects in agriculture as biocontrol agents against plant pathogens. They produce a wide range of secondary metabolites with antifungal, antibacterial, and enzymatic activities, making them valuable in biological control. However, *T. viride* can also be a contaminant in various environments, including indoor spaces and industrial processes, where it can cause the spoilage and degradation of materials. The ability of *Trichoderma* species to develop resistance to chemical fungicides necessitates the exploration of alternative antimicrobial agents, such as essential oils, to manage and control their growth effectively [56].

The antimicrobial activity of *H. italicum* essential oil against *Trichoderma viride* was evaluated in two studies (Table 21), revealing significant variability in its efficacy depending on the strain and origin of the isolates tested. Namely, Dzamic et al. [17] investigated the essential oil’s activity against *Trichoderma viride* IAM 5061. They reported a Minimal Inhibitory Concentration (MIC) value of 14.50 mg/mL and a Minimal Bactericidal Concentration (MBC) value of 48.35 mg/mL. These findings suggest that while the essential oil can inhibit the growth of this strain at a relatively moderate concentration, a higher concentration is required to achieve a bactericidal effect. On the other hand, Stupar et al. [25] examined the antimicrobial properties of the essential oil against a culture heritage isolate of *T. viride* from wood. They found that both the MIC and MBC values exceeded 100 µL/mL, indicating a much higher resistance of this isolate to the essential oil compared to the IAM 5061 strain. This significant difference in sensitivity highlights the importance of strain-specific responses and suggests that the efficacy of *H. italicum* essential oil can vary widely among different isolates of the same species.

#### 3.4.5. Other Fungi

Other fungi were used in testing the antifungal activity of *H. italicum* essential oil, and 13 strains were mentioned only once: *Alternaria alternata*, *Ascohyta rabei*, *Bipolaris spicifera*, *Botrytis cinerea*, *Cercospora beticola*, *Epicoccum nigrum*, *Helminthosporium oryzae*, *Phytophthora capsica*, *Pyricularia oryzae*, *Pythium ultimum*, *Rhizoctonia solani*, *Sclerotium rolfsii*, and *Septoria tritici* (Table 22).

In brief, Djihane et al. [21] reported that the MIC and MBC values for *Alternaria alternata* and *Ascochyta rabiei* were 25.3 μg/mL and 50.6 μg/mL, respectively, indicating high sensitivity to the essential oil. Similarly, Stupar et al. [25] found that *Bipolaris spicifera*, a culture heritage isolate from stone, had higher MIC and MBC values of 75 μL/mL and 100 μL/mL, respectively, showing moderate sensitivity. Angioni et al. [27] examined several fungi, finding that *Botrytis cinerea*, *Helminthosporium oryzae*, and *Rhizoctonia solani* exhibited very high resistance, with MIC values greater than 1000 mg/mL. *Cercospora beticola* showed a MIC range of 500–1000 mg/mL, indicating some variability in sensitivity depending on the essential oil sample used. Additionally, they reported that *Phytophthora capsici* had a MIC range of 125–500 mg/mL, *Pyricularia oryzae* ranged from 500 to 1000 mg/mL, and *Sclerotium rolfsii* had a MIC range of 62–125 mg/mL, highlighting the differential responses among various fungi. Stupar et al. [25] also noted that *Epicoccum nigrum*, another culture heritage isolate from stone, had MIC and MBC values of 25 μL/mL and 50 μL/mL, respectively, indicating higher sensitivity compared to *Bipolaris spicifera*.

The testing of various fungi, including those listed above, in the context of antimicrobial resistance is critical due to the significant impacts that these pathogens can have on human health, agriculture, and the environment. Many fungi, such as *Alternaria alternata* and *Bipolaris spicifera*, can cause opportunistic infections in humans, particularly in immunocompromised individuals [57]. Fungi like *Phytophthora capsici* and *Rhizoctonia solani* are notorious plant pathogens that can cause substantial crop losses and threaten food security [58]. Fungi such as *Epicoccum nigrum* and *Sclerotium rolfsii* can contaminate various environments, leading to the spoilage and degradation of materials. Understanding their sensitivity to essential oils can help develop better control strategies to maintain environmental hygiene [59].

## 4. Main Observations of Antibacterial and Antifungal Potentials of *H. italicum* Essential Oils

The antimicrobial efficacy of *H. italicum* essential oils varies significantly across different microorganisms. The most sensitive microorganisms generally include Gram-positive bacteria such as *Staphylococcus aureus* and *Bacillus cereus*, while Gram-negative bacteria like *Escherichia coli* and *Pseudomonas aeruginosa* tend to be more resistant. Fungi and yeasts, such as *Candida albicans*, also show varying degrees of sensitivity depending on the essential oil composition.

The antimicrobial activity of *H. italicum* essential oils is closely linked to their chemical composition. Essential oils high in neryl acetate exhibit strong antimicrobial activity, particularly against *Staphylococcus aureus* and *Candida albicans*. These oils are also effective against some Gram-negative bacteria, although their efficacy is generally lower compared to Gram-positive strains. Oils rich in γ-curcumene have shown significant effectiveness against *Bacillus cereus* and *Escherichia coli*. The presence of γ-curcumene is associated with broad-spectrum antimicrobial properties, including activity against both bacteria and fungi. α-Pinene-rich essential oils are particularly effective against *Pseudomonas aeruginosa* and *Staphylococcus epidermidis.* These oils exhibit moderate-to-high antimicrobial activity across various microorganisms, suggesting a versatile application in combating microbial infections. In general, *H. italicum* essential oils demonstrate greater efficacy against Gram-positive bacteria compared to Gram-negative bacteria. This is likely due to the structural differences in their cell walls, with Gram-positive bacteria being more susceptible to the disruption caused by essential-oil constituents. The oils also exhibit notable antifungal activity, particularly against yeasts such as *Candida albicans*, highlighting their potential use in treating fungal infections. The origin of the plant material significantly influences the composition and, consequently, the antimicrobial activity of the essential oils. For instance, *H. italicum* oils from Corsica, known for their high neryl acetate content, show different antimicrobial profiles compared to oils from Bosnia, which are richer in α-pinene and γ-curcumene [60]. The mode of preparation, particularly hydrodistillation, plays a crucial role in determining the essential oil’s composition. Factors such as particle size, distillation time, and temperature can affect the yield and proportions of bioactive compounds. The studies reviewed utilized mainly hydrodistillation or steam distillation (commercial samples), but variations in methodology could lead to differences in antimicrobial efficacy. It is critical to standardize these parameters to ensure consistency and reliability in the results. From a global perspective, *H. italicum* essential oils offer a promising natural alternative to synthetic antimicrobials. Their broad-spectrum activity, particularly against Gram-positive bacteria and fungi, positions them as valuable agents in combating microbial resistance. However, the variability in composition and preparation methods underscores the need for standardized protocols to maximize their therapeutic potential.

## 5. Conclusions

The presented review on the antimicrobial potential of *H. italicum* (Roth) G. Don essential oil provides comprehensive insights into its effectiveness against a variety of pathogens, highlighting its broad-spectrum antimicrobial properties. However, several critical aspects need to be considered for a balanced perspective. First, while the review aggregates data from multiple studies, the variability in antimicrobial activity across different strains and compositions of essential oil suggests a need for standardized testing methods. The influence of geographical and botanical variations on the chemical composition of the oil adds another layer of complexity, indicating that not all essential oils from this plant will have uniform efficacy. Additionally, the study focuses heavily on in vitro studies, which, although promising, do not always translate directly to in vivo efficacy due to differences in biological environments. Future research should address these variability issues by standardizing protocols for obtaining essential oil and testing protocols to provide more consistent results. Investigations should also extend beyond in vitro studies to include in vivo models, which can offer more relevant insights into the practical applications of *H. italicum* essential oil in clinical and agricultural settings. There is also a need to explore the synergistic effects of the oil with conventional antibiotics, which could help in combating antimicrobial resistance. Furthermore, understanding the mechanisms of action at the molecular level could provide valuable insights into enhancing the antimicrobial properties of the oil through targeted modifications or combinations with other bioactive compounds. Formulations incorporating *H. italicum* essential oil should be developed and tested for various applications, such as topical treatments, wound dressings, and natural preservatives in food and cosmetic products.

## Figures and Tables

**Table 1:** Antimicrobial potential of *H. italicum* essential oil (papers are listed from newest to oldest).

Reference	Origin	Chemical Composition	Antimicrobial Test	Tested Microorganism
[9]	Serbia	γ-curcumene (13.11–19.98%), α-pinene (9.75–14.45%), ar-curcumene (5.8–14.0%)	Agar well diffusion	B: *Esherichia coli*, *Pseudomonas aeruginosa*, *Salmonella* Typhimurium, *Staphyloccocus aureus*, *Bacillus cereus*, *Listeria monocytogenes* Y: *Saccharomyces cerevisiae*, *Candida albicans* F: *Penicillium aurantiogriseum*, *Aspergillus brasiliensis*
[10]	Serbia	neryl acetate (15.6%), α-pinene (11.5%), γ-curcumene (11.3%)	Agar well diffusion and broth microdilution	B: *S. aureus*, *Bacillus subtilis*, *B. cereus*, *P. aeruginosa*, *E. coli*
[11]	Hungary	neryl acetate (21.2%), ar-curcumene (15.9%)	Broth microdilution	B: *P. aeruginosa*, *Streptococcus pneumoniae*, *Haemophilus influenzae*, *Haemophilus parainfluenzae*
[12]	Poland	neryl acetate (16.4–20.3%), nerol (4.5–15.7%), α-pinene (4.1–10.4%)	Broth microdilution	B: *E. coli*, *P. aeruginosa*, *S. aureus*
[13]	Serbia	γ-curcumene (13.6%), β-selinene (12.2%), α-pinene (11.8%)	Broth microdilution	B: *E. coli*, *P. aeruginosa*, *Salmonella* Enteritidis, *S.* Typhimurium, *Klebsiella aerogenes*, *Proteus hauseri*, *Bacillus spizizenii*, *B. cereus*, *Enterococcus faecalis*, *L. monocytogenes*, *L. innocua*, *L. ivanovii*, *Rhodococcus equi*, *Staphylococcus epidermidis*, *S. aureus*
[14]	Commercial oils of different origins; Belgium, France, Serbia	neryl acetate (9.2–35.0%), α-pinene (1.7–19.9%), γ-curcumene (10.0–16.8%)	Broth microdilution	B: *B. cereus*, *E. faecalis*, *Sarcina lutea*, *S. aureus*, *S. epidermidis*, *Acinetobacter baumannii*, *Enterobacter aerogenes*, *E. coli*, *S.* Enteritidis Y: *C. albicans*
[15]	Bulgaria	neryl acetate (3.8–33.9%), α-pinene (1.9–20.8%), γ-curcumene (8.8–16.5%)	Agar well diffusion	B: *S. aureus*, *B. subtilis*, *E. coli*, *P. aeruginosa* Y: *S. cerevisiae*, *C. albicans* F: *A. brasiliensis*, *Fusarium moniliforme*
[16]	Montenegro	β-eudesmene (21.7%), α-pinene (16.9%), β-caryophyllene (11.5%), neryl acetate (10.7%)	Broth microdilution and vapor phase assay	B: *S. aureus*, *K. pneumoniae*, *E. coli*, *A. baumannii*, *P. aeruginosa* Y: *C. albicans*
[17]	Croatia	neryl acetate (20.4%), γ-curcumene (14.1%)	Broth microdilution	B: *B. cereus*, *E. coli*, *L. monocytogenes*, *P. aeruginosa*, *S.* Typhimurium, *S. aureus* Y: *C. albicans* F: *Asperigullus fumigatus*, *A. versicolor*, *A. ochraceus*, *A. niger*, *Trichoderma viride*, *Penicillium funiculosum*, *P. ochrochloron*
[18]	Montenegro	γ-curcumene (14.1%), β-selinene (11.3%), ar-curcumene (10.4%)	Broth microdilution	B: *E. coli*, *S. aureus*, *B. subtilis*, *S.* Enteritidis, *L. monocytogenes*, *Proteus mirabilis*, *P. aeruginosa*, *E. faecalis* Y: *C. albicans*
[19]	Croatia	α-pinene (21.6%), γ-curcumene (21.6%)	Agar well diffusion and Broth microdilution	B: *S. aureus*, *S. epidermidis*, *A. baumannii*, *P. aeruginosa*, *E. coli* Y: *C. albicans*
[20]	Croatia	α-pinene (21.6%), γ-curcumene (21.6%)	Broth microdilution	B: *Mycobacterium avium*, *M. intracellulare*
[21]	Algeria	α-cedrene (13.6%), ar-curcumene (11.4%), geranyl acetate (10.1%)	Agar well diffusion	B: *E. coli*, *S. aureus*, *Micrococcus luteus*, *K. pneumoniae*, *Enterococcus cereus*, *B. cereus*, *S. epidermidis*, *B. subtilis*, *P. aeruginosa*, *E. faecalis*, *P. mirabilis*, *L. monocytogenes* Y: *Candida albicans*, *Saccharomyces cerevisiae*, F: *Fusarium solani* var. *coeruleum*, *Aspergilus niger*, *Alternaria alternata*, *Ascochyta rabiei*
[22]	Algeria	isopropyl tetradecanoate (12.1), α-pinene (12.0%)	Agar well diffusion	B: *Shigella* sp., *E. coli*, *K. pneumoniae*, *S. aureus*, *Pseudomonas syringae* Y: *C. albicans*
[23]	France	neryl acetate (33.8%), γ-curcumene (14.2%)	Broth microdilution	B: *S. aureus*
[24]	France	neryl acetate (32.7%), γ-curcumene (11.6%)	Broth microdilution	B: *E. coli*, *S. aureus*, *B. subtilis*, *S.* Typhimurium, *K. pneumoniae*, *P. aeruginosa*, *Bacillus pumilus*
[25]	Serbia	γ-curcumene (22.5%), α-pinene (15.9%)	Vapor phase assay (micro-atmosphere conditions)	F: *A. niger*, *A. ochraceus*, *Bipolaris spicifera*, *Epicoccum nigrum*, *Penicillium* sp., *Trichoderma viride*
[26]	Croatia	neryl acetate (11.5%), α-pinene (10.2%),	Agar well diffusion and Broth microdilution	B: *S. aureus*, *E. coli*, *P. aeruginosa* Y: *C. albicans*
[27]	Italy	linalool (11.3–17.3%), neryl acetate (23.6–31.1%), γ-curcumene (12.2–14.4%)	Agar well diffusion	F: *Botrytis cinerea*, *Cercospora beticola*, *Fusarium oxysporum lycopersici*, *F. graminearum*, *Helminthosporium oryzae*, *Pythium ultimum*, *Pyricularia oryzae*, *Rhizoctonia solani*, *Sclerotium rolfsii*, *Phytophthora capsici*, *Septoria tritici*
[28]	Greece	geraniol (35.6%), geranyl acetate (14.7%), nerolidol (11.9%)	Broth microdilution	B: *S. aureus*, *S. epidermidis*, *P. aeruginosa*, *K. pneumoniae*, *E. cloaceae*, *E. coli*

B—bacteria; Y—yeast; F—fungi.

**Table 2:** A detailed analysis of the antimicrobial activity of the essential oil of *H. italicum* on *S. aureus*.

Reference	*S. aureus* Strain	Inhibition Zone Diameters (mm)	MIC Value(s)	MBC Value(s)
[9]	ATCC 25923	10.00–19.67 *^;1^	/	/
[10]	ATCC 25923	10.00 ^2^	5 µg/mL	/
[12]	ATCC 25923	/	1–4 mg/mL *	16 mg/mL
	MRSA	/	4–8 mg/mL *	32 mg/mL
[13]	ATCC 25923	/	>454.5 µg/mL	>454.5 µg/mL
[14]	ATCC 6538	/	0.30–10.00 mg/mL *	/
[15]	ATCC 6538	8.00–11.30 *^;3^	/	/
[16]	ATCC 29213	/	>5%	>5%
[17]	ATCC 6538	/	15 mg/mL	>15 mg/mL
[18]	ATCC 25923	/	<1.4 µg/mL	1.4 µg/mL
[19]	ATCC 25923	8.50 ^2^	1.6 mg/mL	3.2 mg/mL
	Clinical MRSA isolate	7.50 ^2^	6.4 mg/mL	12.8 mg/mL
[21]	ATCC 6538	27.00 ^1^	50.6 µg/mL	50.6 µg/mL
[22]	ATCC 25923	10.00 ^2^	/	/
[23]	ATCC 25923	/	0.5 mg/mL	1.00 mg/mL
[24]	ATCC 25923	/	0.05%	0.05%
[26]	ATCC 25923	10.00 ^4^	5 µL/mL	/
[28]	ATCC 25923	/	3.25 mg/mL	/

MIC—Minimal Inhibitory Concentration; MBC—Minimal Bactericidal Concentration; ATCC—American Type Culture Collection; MRSA—methicillin resistant *S. aureus*; * depend on essential oil sample. Concentration of essential oil in the observed zone: ^1^ 15 μL; ^2^ serial dilution, ^3^ 50 μL, ^3^ 10 μL, and ^4^ 5 μL.

**Table 3:** A detailed analysis of the antimicrobial activity of the essential oil of *H. italicum* on *S. epidermidis*.

Reference	*S. epidermidis* Strain	Inhibition Zone Diameters (mm)	MIC Value (s)	MBC Value (s)
[13]	ATCC 12228	/	454.5 µg/mL	454.5 µg/mL
[14]	ATCC 12228	/	0.60–2.50 mg/mL *	/
[19]	Clinical isolate	7.50 ^1^	6.4 mg/mL	6.4 mg/mL
	Clinical MRSE isolate	8.00 ^1^	12.8 mg/mL	12.8 mg/mL
[21]	ATCC 12228	23.00 ^2^	25.3 µg/mL	25.3 µg/mL
[28]	ATCC 12228	/	3.25 mg/mL	/

MIC—Minimal Inhibitory Concentration; MBC—Minimal Bactericidal Concentration; ATCC—American Type Culture Collection; MRSE—methicillin resistant *S. epidermidis*; * depend on essential oil sample. Concentration of essential oil in the observed zone: ^1^ serial dilution and ^2^ 15 μL.

**Table 4:** A detailed analysis of the antimicrobial activity of the essential oil of *H. italicum* on *B. cereus*.

Reference	*B. cereus* Strain/Origin	Inhibition Zone Diameters (mm)	MIC Value (s)	MBC Value (s)
[9]	ATCC 11778	n.d.–12.67 *^;1^	/	/
[10]	ATCC 14579	13.00 ^2^	10.00 μg/mL	/
[13]	ATCC 11778	/	454.50 μL/mL	454.50 μL/mL
[14]	ATCC 11778	/	1.2–10 mg/mL *	/
[17]	Clinical isolate	/	2.5 mg/mL	>2.5 mg/mL
[21]	ATCC 10876	/	3.162 μL/mL	12.65 μL/mL

MIC—Minimal Inhibitory Concentration; MBC—Minimal Bactericidal Concentration; ATCC—American Type Culture Collection; * depend on essential oil sample; n.d.—not detected. Concentration of essential oil in the observed zone: ^1^ 15 μL and ^2^ serial dilution.

**Table 5:** A detailed analysis of the antimicrobial activity of the essential oil of *H. italicum* on *B. subtilis*.

Reference	*B. subtilis* Strain	Inhibition Zone Diameters (mm)	MIC Value (s)	MBC Value (s)
[10]	ATCC 19659	12.00 ^1^	10.00 μg/mL	/
[15]	ATCC 6633	8.00–9.20 *^;2^	/	/
[18]	ATCC 6633	/	1.4 μg/mL	1.4 μg/mL
[21]	ATCC 9372	/	12.65 μg/mL	50.6 μg/mL
[24]	ATCC 6633	/	0.05%	0.1%

MIC—Minimal Inhibitory Concentration; MBC—Minimal Bactericidal Concentration; ATCC—American Type Culture Collection; * depend on essential oil sample; Concentration of essential oil in the observed zone: ^1^ serial dilution and ^2^ 50 μL.

**Table 6:** A detailed analysis of the antimicrobial activity of the essential oil of *H. italicum* on *L. monocytogenes*.

Reference	*L. monocytogenes* Strain	Inhibition Zone Diameters (mm)	MIC Value (s)	MBC Value (s)
[9]	ATCC 19115	7.33–17.00 *^;1^	/	/
[13]	ATCC 19111	/	454.50 μg/mL	454.50 μg/mL
[17]	NCTC 7973	/	>15.00 mg/mL	>15.00 mg/mL
[18]	ATCC 19111	/	<1.4 μg/mL	1.4 μg/mL
[21]	ATCC 15313	/	n.d.	n.d.

MIC—Minimal Inhibitory Concentration; MBC—Minimal Bactericidal Concentration; ATCC—American Type Culture Collection; NCTC—National Collection of Type Cultures (UK); * depend on essential oil sample; n.d.—not detected. Concentration of essential oil in the observed zone: ^1^ 15 μL.

**Table 7:** A detailed analysis of the antimicrobial activity of the essential oil of *H. italicum* on *E. faecalis*.

Reference	*E. faecalis* Strain	Inhibition Zone Diameters (mm)	MIC Value (s)	MBC Value (s)
[13]	ATCC 29212	/	>454.50 μg/mL	>454.50 μg/mL
[14]	ATCC 19433	/	2.50–10.00 mg/mL *	/
[18]	ATCC 19433	/	7.1 μg/mL	14.0 μg/mL
[21]	ATCC 49452	/	50.6 μg/mL	101.2 μg/mL

MIC—Minimal Inhibitory Concentration; MBC—Minimal Bactericidal Concentration; ATCC—American Type Culture Collection; * depend on essential oil sample.

**Table 8:** A detailed analysis of the antimicrobial activity of the essential oil of *H. italicum* on other Gram-positive bacteria.

Reference	Bacteria	Strain	Inhibition Zone Diameters (mm)	MIC Value (s)	MBC Value (s)
[11]	*Streptococcus pneumoniae*	DSM 20566	/	0.375 mg/mL	/
[13]	*Listeria innocua*	ATCC 33090	/	>454.50 μg/mL	>454.50 μg/mL
	*Listeria ivanovii*	ATCC 19119	/	>454.50 μg/mL	>454.50 μg/mL
	*Bacillus spizizenii*	ATCC 6633	/	>454.50 μg/mL	>454.50 μg/mL
	*Rhodococcus equi*	ATCC 6939	/	454.50 μg/mL	454.50 μg/mL
[14]	*Sarcina lutea*	ATCC 9431	/	2.5–10.0 mg/mL *	/
[20]	*Mycobacterium avium*	ATCC 25291	/	3.2 mg/mL	3.2 mg/mL
	*Mycobacterium intracellulare*	ATCC 13950	/	3.2 mg/mL	3.2 mg/mL
[21]	*Enterococcus cereus*	ATCC 2035	/	0.79 μg/mL	μg/mL
	*Micrococcus luteus*	ATCC 4698	/	6.325 μg/mL	12.65 μg/mL
[24]	*Bacillus pumilus*	ATCC 27142	/	0.05%	0.10%

MIC—Minimal Inhibitory Concentration; MBC—Minimal Bactericidal Concentration; DSM—German Collection of Microorganisms and Cell Cultures GmbH; ATCC—American Type Culture Collection; * depend on essential oil sample.

**Table 9:** A detailed analysis of the antimicrobial activity of the essential oil of *H. italicum* on *E. coli*.

Reference	*E. coli* Strain	Inhibition Zone Diameters (mm)	MIC Value (s)	MBC Value (s)
[9]	ATCC 25922	n.d. ^1^	/	/
[10]	ATCC 14169	n.d. ^2^	n.d.	/
[12]	ATCC 25922	/	32.0–64.0 mg/mL *	>64 mg/mL
[13]	ATCC 8739	/	>0.4545 µg/mL	>0.4545 µg/mL
	ATCC 10536	/	>0.4545 µg/mL	>0.4545 µg/mL
[14]	ATCC 25922	/	2.50–10.00 mg/mL *	/
[15]	ATCC 8739	8.00 ^3^	/	/
[16]	ATCC 25922	/	>5% **	>5% **
[17]	ATCC 35210	/	2.50 mg/mL	2.50 mg/mL
[18]	ATCC 25922	/	n.d.	/
[19]	ATCC 25922	6.00 ^2^	12.80 mg/mL	12.80 mg/mL
	ESBL+ clinical isolate	6.00 ^2^	12.80 mg/mL	12.80 mg/mL
[21]	ATCC 25922	n.d. ^1^	n.d.	n.d.
[22]	ATCC 25922	14.00 ^2^	/	/
[24]	ATCC 25922	/	0.05%	0.05%
[26]	ATCC 25922	66.00 ^4^	7.00 µL/mL	/
[28]	ATCC 25922	/	n.d.	/

MIC—Minimal Inhibitory Concentration; MBC—Minimal Bactericidal Concentration; ATCC—American Type Culture Collection; ESBL—extended-spectrum beta-lactamase; * depend on essential oil sample; n.d.—not detected; ** % of the initial concentration of the essential oil. Concentration of essential oil in the observed zone: ^1^ 15 μL, ^2^ serial dilution, ^3^ 50 μL, and ^4^ 5 μL.

**Table 10:** A detailed analysis of the antimicrobial activity of the essential oil of *H. italicum* on *P. aeruginosa*.

Reference	*P. aeruginosa* Strain	Inhibition Zone Diameters (mm)	MIC Value (s)	MBC Value (s)
[9]	ATCC 27853	n.d. ^1^	/	/
[10]	ATCC 27853	10.00 ^2^	5.00 µg/mL	/
[11]	ATCC 27853	/	0.625 mg/mL	/
[12]	ATCC 27853	/	32.00–64.00 mg/mL *	>64.00 mg/mL
[13]	ATCC 27853	/	>0.4545 µg/mL	>0.4545 µg/mL
[15]	ATCC 9027	8.00 ^3^	/	/
[16]	CR-Pa	/	>5%	>5%
[17]	ATCC 27853	/	1.5 mg/mL	>1.5 mg/mL
[18]	ATCC 27853	/	n.d.	/
[19]	ATCC 27853	6.00 ^2^	12.80 mg/mL	12.80 mg/mL
[21]	ATCC 27853	/	12.56 µg/mL	12.56 µg/mL
[24]	ATCC 27853	/	0.05%	0.1%
[26]	ATCC 27853	66.00 ^4^	/	/
[28]	ATCC 27853	/	3.75 mg/mL	/

MIC—Minimal Inhibitory Concentration; MBC—Minimal Bactericidal Concentration; ATCC—American Type Culture Collection; CR-Pa—carbapenem-resistant *P. aeruginosa*; * depend on essential oil sample; n.d.—not detected. Concentration of essential oil in the observed zone: ^1^ 15 μL and ^2^ serial dilution, ^3^ 50 μL, and ^4^ 5 μL.

**Table 11:** A detailed analysis of the antimicrobial activity of the essential oil of *H. italicum* on *S.* Typhimurium and Enteritidis.

Reference	*S. enterica* Strain	Inhibition Zone Diameters (mm)	MIC Value (s)	MBC Value (s)
[9]	ATCC 13311	n.d. ^1^	/	/
[13]	ATCC 13076	/	>454.50 µg/mL	>454.50 µg/mL
	ATCC 14028	/	>454.50 µg/mL	>454.50 µg/mL
[14]	ATCC 13076	/	5.0–10.0 mg/mL *	/
[17]	ATCC 13311	/	2.5 mg/mL	2.5 mg/mL
[18]	ATCC 13076	/	n.d.	n.d.
[24]	ATCC 19659	/	0.05%	0.10%

MIC—Minimal Inhibitory Concentration; MBC—Minimal Bactericidal Concentration; ATCC—American Type Culture Collection; * depend on essential oil sample; n.d.—not detected. Concentration of essential oil in the observed zone: ^1^ 15 μL.

**Table 12:** A detailed analysis of the antimicrobial activity of the essential oil of *H. italicum* on *K. pneumoniae*.

Reference	*K. pneumoniae* Strain/Origin	Inhibition Zone Diameters (mm)	MIC Value (s)	MBC Value (s)
[16]	CR-Kp	/	>5.0%	>5.0%
[21]	ATCC 4352	/	n.d.	n.d.
[22]	ATCC 700603	13.00 ^1^	/	/
[24]	ATCC 13883	/	0.05%	0.1%
[28]	ATCC 13883	/	3.50 mg/mL	/

MIC—Minimal Inhibitory Concentration; MBC—Minimal Bactericidal Concentration; ATCC—American Type Culture Collection; CR-Kp—carbapenem-resistant *K. pneumoniae*; n.d.—not detected. Concentration of essential oil in the observed zone: ^1^ serial dilution.

**Table 13:** A detailed analysis of the antimicrobial activity of the essential oil of *H. italicum* on *A. baumannii*.

Reference	*A. baumannii* Strain	Inhibition Zone Diameters (mm)	MIC Value (s)	MBC Value (s)
[14]	ATCC 19606	/	1.2–5.0 µg/mL *	/
[16]	CR-Ab isolate	/	5%	5%
[19]	ATCC BAA-1605	14.00 ^1^	3.2 mg/mL	3.2 mg/mL
	ATCC 19606	13.50 ^1^	3.2 mg/mL	3.2 mg/mL

MIC—Minimal Inhibitory Concentration; MBC—Minimal Bactericidal Concentration; ATCC—American Type Culture Collection; CR—carbapenem-resistant *A. baumannii*; * depend on essential oil sample. Concentration of essential oil in the observed zone: ^1^ serial dilution.

**Table 14:** A detailed analysis of the antimicrobial activity of the essential oil of *H. italicum* on *P. mirabilis*.

Reference	*P. mirabilis* Strain	Inhibition Zone Diameters (mm)	MIC Value (s)	MBC Value (s)
[18]	ATCC25933	/	n.d.	n.d.
[21]	ATCC 35659	/	1.581 µg/mL	3.162 µg/mL

MIC—Minimal Inhibitory Concentration; MBC—Minimal Bactericidal Concentration; ATCC—American Type Culture Collection; n.d.—not detected.

**Table 15:** A detailed analysis of the antimicrobial activity of the essential oil of *H. italicum* on other Gram-negative bacteria.

Reference	Bacteria	Strain	Inhibition Zone Diameters (mm)	MIC Value (s)	MBC Value (s)
[11]	*Haemophilus influenzae*	DSM 4690	/	0.312 mg/mL	/
	*Haemophilus parainfluenzae*	DSM 8978	/	0.312 mg/mL	/
	*Streptococcus pneumoniae*	DSM 20566	/	0.375 mg/mL	/
[13]	*Klebsiella aerogenes*	ATCC 13048	/	>454.50 µg/mL	>454.50 µg/mL
	*Proteus hauseri*	ATCC 13315	/	>454.50 µg/mL	>454.50 µg/mL
[14]	*Enterobacter aerogenes*	ATCC 13048	/	2.5–5.0 mg/mL *	/
[22]	*Pseudomonas syringae*	N/A	9.00 ^1^	/	/
	*Shigella* sp.	N/A	13.00 ^1^	/	/
[28]	*Enterobacter cloacae*	ATCC 13047	/	3.5 mg/mL	/

MIC—Minimal Inhibitory Concentration; MBC—Minimal Bactericidal Concentration; ATCC—American Type Culture Collection; DSM—German Collection of Microorganisms and Cell Cultures GmbH; * depend on essential oil sample; N/A—not applicable. Concentration of essential oil in the observed zone: ^1^—Serial dilution.

**Table 16:** A detailed analysis of the antimicrobial activity of the essential oil of *H. italicum* on *C. albicans*.

Reference	Strain	Inhibition Zone Diameters (mm)	The MIC Values	The MFC Values
[9]	ATCC 10231	7.67–13.67 *^;1^	/	/
[14]	ATCC 10231	/	2.50–5.00 mg/mL *	/
[15]	ATCC 10231	8.00 ^2^	/	/
[16]	ATCC 14053	/	5.00%	5.00%
[17]	ATCC 10231	/	24.17 mg/mL	24.17 mg/mL
[18]	ATCC 10231	/	n.d.	n.d.
[19]	ESBL+ clinical isolate	7.50 ^3^	6.4 mg/mL	n.d.
[21]	ATCC 10231	/	6.325 μg/mL	12.65 μg/mL
[22]	N/A	n.d. ^3^	/	/
[26]	ATCC 10231	9.00 ^4^	5.00 μL/mL	/

MIC—Minimal Inhibitory Concentration; MFC—Minimal Fungicide Concentration; ATCC—American Type Culture Collection; ESBL—extended spectrum beta-lactamase; * depend on essential oil sample; N/A—not applicable; n.d.—not detected. Concentration of essential oil in the observed zone: ^1^ 15 μL, ^2^ 50 μL, ^3^ serial dilution, and ^4^ 5 μL.

**Table 17:** A detailed analysis of the antimicrobial activity of the essential oil of *H. italicum* on *S. cerevisiae*.

Reference	Strain/Origin	Inhibition Zone Diameters (mm)	MIC Value (s)	MBC Value (s)
[9]	ATCC 9763	13.67–18.67 *^;1^	/	/
[15]	ATCC 9763	8.00 ^2^	/	/
[21]	ATCC 9763	/	6.325 μg/mL	12.65 μg/mL

MIC—Minimal Inhibitory Concentration; MBC—Minimal Bactericidal Concentration; ATCC—American Type Culture Collection; * depend on essential oil sample. Concentration of essential oil in the observed zone: ^1^ 15 μL and ^2^ 50 μL.

**Table 18:** A detailed analysis of the antimicrobial activity of the essential oil of *H. italicum* on *Aspergiullus* species.

Reference	*Aspergillus* Species	Strain/Origin	Inhibition Zone Diameters (mm)	MIC Value (s)	MBC Value (s)
[9]	*Aspergillus brasiliensis*	ATCC 16404	n.d. ^1^	/	/
[15]	*Aspergillus brasiliensis*	ATCC 16404	17.41–18.00 *^;2^	/	/
[17]	*Aspergillus fumigatus*	Clinical isolate	/	14.50 mg/mL	48.35 mg/mL
	*Aspergillus niger*	ATCC 6275	/	14.50 mg/mL	48.35 mg/mL
	*Aspergillus ochraceus*	ATCC 12066	/	14.50 mg/mL	48.35 mg/mL
	*Aspergillus versicolor*	ATCC 11730	/	14.50 mg/mL	48.35 mg/mL
[21]	*Aspergillus niger*	Internal collection **	/	25.3 μg/mL	50.6 μg/mL
[25]	*Aspergillus niger*	Culture heritage isolate (wood)	/	75 μL/mL	100 μL/mL
	*Aspergillus ochraceus*	Culture heritage isolate (wood)	/	75 μL/mL	100 μL/mL

MIC—Minimal Inhibitory Concentration; MBC—Minimal Bactericidal Concentration; ATCC—American Type Culture Collection; ** Laboratory of Applied Microbiology, Faculty of Nature and Life Sciences, University Ferhat Abbas, Setif; * depend on essential oil sample; n.d.—not detected. Concentration of essential oil in the observed zone: ^1^—15 μL; ^2^—50 μL.

**Table 19:** A detailed analysis of the antimicrobial activity of the essential oil of *H. italicum* on *Penicillium* species.

Reference	*Penicillium* Species	Strain/Origin	Inhibition Zone Diameters (mm)	MIC Value (s)	MBC Value (s)
[9]	*P. aurantiogriseum*	ATCC 16025	n.d. ^1^	/	/
[17]	*P. funiculosum*	ATCC 36839	/	14.50 mg/mL	24.17 mg/mL
	*P. ochrochloron*	ATCC 9112	/	14.50 mg/mL	24.17 mg/mL
[25]	*Penicillium* sp.	culture heritage isolate (wood)	/	25 μL/mL	50 μL/mL

MIC—Minimal Inhibitory Concentration; MBC—Minimal Bactericidal Concentration; ATCC—American Type Culture Collection; n.d.—not detected. Concentration of essential oil in the observed zone: ^1^ 15 μL.

**Table 20:** A detailed analysis of the antimicrobial activity of the essential oil of *H. italicum* on *Fusarium* species.

Reference	*Fusarium* Species	Strain/Origin	Inhibition Zone Diameters (mm)	MIC Value (s)	MBC Value (s)
[15]	*F. moniliforme*	Clinical isolate	8.00 ^1^	/	/
[21]	*F. solani* var. *coeruleum*	internal collection **	/	50.6 μg/mL	50.6 μg/mL
[27]	*F. graminearum*	N/A	/	500–1000 * mg/mL	/
	*F. oxysporum* var. *lycopersici*	N/A	/	>1000 mg/mL	>1000 mg/mL

MIC—Minimal Inhibitory Concentration; MBC—Minimal Bactericidal Concentration; ** Laboratory of Applied Microbiology, Faculty of Nature and Life Sciences, University Ferhat Abbas, Setif; * depend on essential oil sample; N/A—not applicable. Concentration of essential oil in the observed zone: ^1^ 50 μL.

**Table 21:** A detailed analysis of the antimicrobial activity of the essential oil of *H. italicum* on *Trihoderma viride*.

Reference	Strain/Origin	Inhibition Zone Diameters (mm)	MIC Value (s)	MBC Value (s)
[17]	IAM * 5061	/	14.50 mg/mL	48.35 mg/mL
[25]	Culture heritage isolate (wood)	/	>100 µL/mL	>100 µL/mL

MIC—Minimal Inhibitory Concentration; MBC—Minimal Bactericidal Concentration; * IAM Collection (University of Tokyo).

**Table 22:** A detailed analysis of the antimicrobial activity of the essential oil of *H. italicum* on other fungi.

Reference	Fungus Species	Strain/Origin	Inhibition Zone Diameters (mm)	MIC Value (s)	MBC Value (s)
[21]	*Alternaria alternata*	Internal collection **	/	25.3 μg/mL	50.6 μg/mL
	*Ascochyta rabiei*	Internal collection **	/	50.6 μg/mL	50.6 μg/mL
[25]	*Bipolaris spicifera*	Culture heritage isolate (stone)	/	75 μL/mL	100 μL/mL
	*Epicoccum nigrum*	Culture heritage isolate (stone)	/	25 μL/mL	50 μL/mL
[27]	*Botrytis cinerea*	N/A	/	>1000 mg/mL	/
	*Cercospora beticola*	N/A	/	500–1000 mg/mL *	/
	*Helminthosporium oryzae*	N/A	/	>1000 mg/mL	/
	*Phytophthora capsici*	N/A	/	125–500 mg/mL	/
	*Pyricularia oryzae*	N/A	/	500–1000 mg/mL *	/
	*Pythium ultimum*	N/A	/	62–250 mg/mL	/
	*Rhizoctonia solani*	N/A	/	>1000 mg/mL	/
	*Sclerotium rolfsii*	N/A	/	62–125 mg/mL *	/
	*Septoria tritici*	N/A	/	125–1000 mg/mL	/

MIC—Minimal Inhibitory Concentration; MBC—Minimal Bactericidal Concentration; ** Laboratory of Applied Microbiology, Faculty of Nature and Life Sciences, University Ferhat Abbas, Setif; * depend on essential oil sample; N/A—not applicable.
